# Ternary structure reveals mechanism of a membrane diacylglycerol kinase

**DOI:** 10.1038/ncomms10140

**Published:** 2015-12-17

**Authors:** Dianfan Li, Phillip J. Stansfeld, Mark S. P. Sansom, Aaron Keogh, Lutz Vogeley, Nicole Howe, Joseph A. Lyons, David Aragao, Petra Fromme, Raimund Fromme, Shibom Basu, Ingo Grotjohann, Christopher Kupitz, Kimberley Rendek, Uwe Weierstall, Nadia A. Zatsepin, Vadim Cherezov, Wei Liu, Sateesh Bandaru, Niall J. English, Cornelius Gati, Anton Barty, Oleksandr Yefanov, Henry N. Chapman, Kay Diederichs, Marc Messerschmidt, Sébastien Boutet, Garth J. Williams, M. Marvin Seibert, Martin Caffrey

**Affiliations:** 1 School of Medicine and School of Biochemistry and Immunology, Trinity College Dublin, Dublin 2, Ireland; 2 Department of Biochemistry, University of Oxford, South Parks Road, Oxford OX1 3QU, UK; 3 School of Molecular Sciences and Center for Applied Structural Discovery at the Biodesign Institute, Arizona State University, Tempe, Arizona 85287, USA; 4 Department of Physics, Arizona State University, Tempe, Arizona 85287, USA; 5 Department of Chemistry, Bridge Institute, University of Southern California, Los Angeles, California 90089, USA; 6 SFI Strategic Research Cluster in Solar Energy Conversion, School of Chemical and Bioprocess Engineering, University College Dublin, Belfield, Dublin 4, Ireland; 7 Center for Free Electron Laser Science, Deutsches Elektronen-Synchrotron, 22607 Hamburg, Germany; 8 Department of Physics, University of Hamburg, 22761 Hamburg, Germany; 9 Department of Biology, University of Konstanz, Box 647, D-78457 Konstanz, Germany; 10 Linac Coherent Light Source (LCLS), SLAC National Accelerator Laboratory, 2575 Sand Hill Road, Menlo Park, California 94025, USA

## Abstract

Diacylglycerol kinase catalyses the ATP-dependent conversion of diacylglycerol to phosphatidic acid in the plasma membrane of *Escherichia coli*. The small size of this integral membrane trimer, which has 121 residues per subunit, means that available protein must be used economically to craft three catalytic and substrate-binding sites centred about the membrane/cytosol interface. How nature has accomplished this extraordinary feat is revealed here in a crystal structure of the kinase captured as a ternary complex with bound lipid substrate and an ATP analogue. Residues, identified as essential for activity by mutagenesis, decorate the active site and are rationalized by the ternary structure. The γ-phosphate of the ATP analogue is positioned for direct transfer to the primary hydroxyl of the lipid whose acyl chain is in the membrane. A catalytic mechanism for this unique enzyme is proposed. The active site architecture shows clear evidence of having arisen by convergent evolution.

## Abstract (web summary)

Diacylglycerol kinase is a small bacterial membrane-bound trimer that catalyses diacylglycerol conversion to phosphatidic acid. Here, the authors solve the crystal structure of the kinase bound to a lipid substrate and an ATP analogue, and show that the active site arose through convergent evolution.

## Graphical abstract

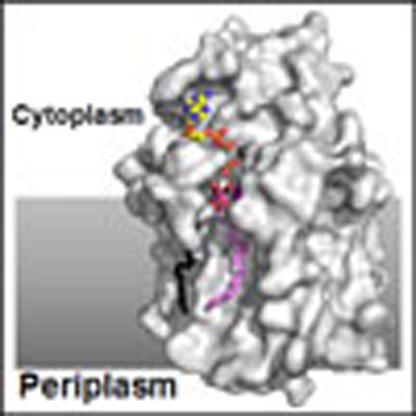

Diacylglycerol kinase (DgkA) is the smallest known kinase. In *Escherichia coli*, it is responsible for the adenosine triphosphate (ATP)-dependent phosphorylation of diacylglycerol (DAG) to phosphatidic acid used in periplasm membrane-derived oligosaccharide synthesis in response to osmotic shock^1^. The enzyme has long served as a model for integral membrane enzymology^1^,^2^,^3^, folding^4^,^5^,^6^, assembly^7^,^8^,^9^ and stability^10^,^11^. It is a trimer with three active sites of the shared sites type that exhibits moderate positive heteroallostery, random-binding kinetics and lipid substrate promiscuity^1^.

High-resolution X-ray structures of wild-type (WT) and functional thermostable mutants (Δ4, Δ7) of DgkA were obtained using protein crystallized in a membrane environment by the lipid cubic phase (LCP) or *in meso* method^12^. In contrast to a solution NMR structure that displayed domain swapping^13^, the crystallized kinase showed a simple quaternary arrangement in which adjacent monomers created shared active sites. Considerable effort was devoted to demonstrating that the enzyme was functionally active in the crystal^12^. While the model included a lipid substrate, a monoacylglycerol (7.8 MAG), its functional relevance remained uncertain in the absence of a nucleotide bound structure. Here we report such a structure, along with mutagenesis studies, molecular dynamics simulations (MDS) and density functional theory (DFT) modelling, which explain a wealth of biochemical and biophysical data and support a mechanism for direct transphosphorylation by this diminutive kinase.

## Results

### Crystallization and overall structure

Because DgkA was crystallized in the LCP composed of MAGs (ref. 14), which double as lipid substrates^15^, solving a structure with ATP bound was impossible – the enzyme turns over catalytically as soon as nucleotide is added. Therefore, the enzyme was co-crystallized with the non-hydrolysable ATP analogue, adenylylmethylenediphosphonate (ACP). Producing structure-grade crystals of the complex involved extensive screening of protein constructs and crystallization conditions including host lipid MAGs. Best crystals, providing a ternary complex structure with ACP and lipid bound at 2.7 Å resolution (Table 1), were obtained with Δ4-DgkA grown in 9.9 MAG at 4 °C and soaked with zinc-ACP.

**Figure 1:**
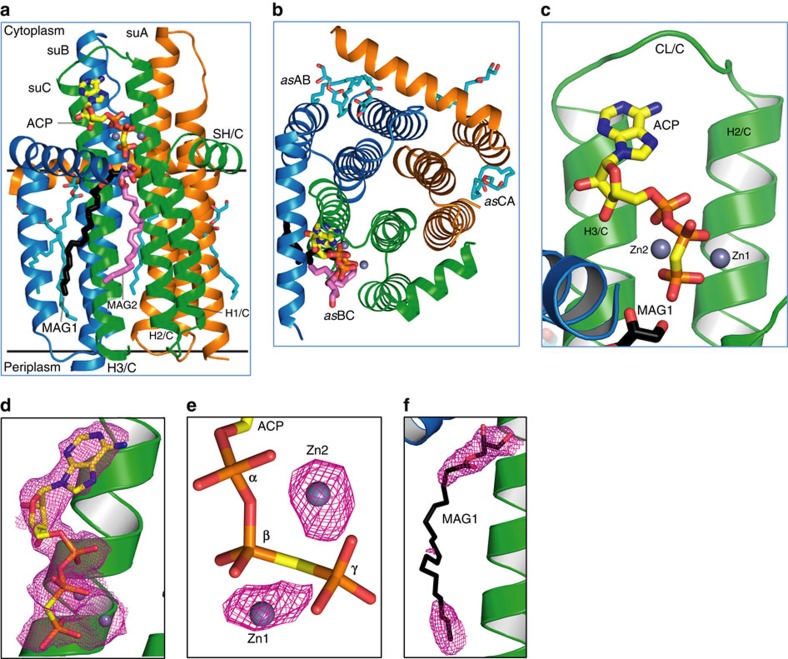
Overall structure of the DgkA-ACP-lipid ternary complex. (**a**,**b**) Views of the kinase trimer parallel to the membrane and from the cytosol, respectively, with subunits represented as brown, blue and green ribbons. ACP and lipid are shown in stick representation. The two grey spheres represent zinc. Putative membrane boundaries are shown as black lines. Active site asBC contains both ACP and lipid substrate. MAG1 and MAG2 are shown with carbons coloured black and violet, respectively. (**c**) Expanded view of zinc-ACP in the binding site. (**d**) Feature enhanced map (Methods section) of Zn-ACP at 1 σ. (**e**) Anomalous density maps for zinc contoured at 4.0 σ. (**f**) Feature enhanced map for lipid MAG1 in asBC contoured at 1 σ.

Like the apo-enzyme^12^, the ternary complex is a homo-trimer (Fig. 1a,b). Each subunit has four helices, three of which are largely membrane embedded (H1-3), whereas the fourth, an N-terminal amphiphilic surface helix (SH), anchors the protein at the membrane interface (Supplementary Fig. 1a). Stereo images of individual subunit models and the electron density on which the models are based are shown in Supplementary Fig. 2. H2 and H3 extend out of the membrane and are connected by a cytosolic loop (CL) whose conformation and sequence position in the protein differ between subunits in ways that may have functional consequences (Supplementary Fig. 3), as discussed below. Individual subunits are identified suA, suB and suC. The trimer has three active sites of the shared sites type. Each site has contributions from two subunits, identified asAB, asBC and asCA, where asAB is created by the SH of suA and H1-3 of suB. In the complex, asBC has zinc-ACP and lipid bound (Fig. 1a,b). The other two sites are nucleotide-free.

### Ternary complex structure

**Figure 2:**
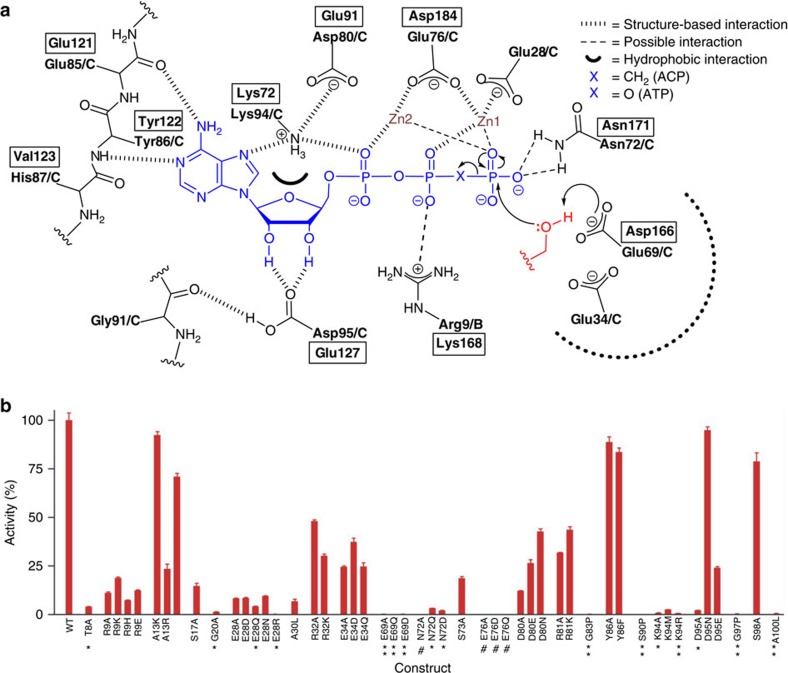
Proposed mechanism and sensitivity to mutagenesis of the transphosphorylation reaction catalysed by DgkA. (**a**) Two-dimensional representation of the active and substrate-binding sites. The catalytic Glu69_C_ abstracts a proton from the primary hydroxyl of lipid substrate MAG1 (red) creating a reactive alkoxide which attacks the γ-phosphorus of zinc-ATP (blue). The transition intermediate includes a pentavalent γ-phosphorus where the two substrates are covalently bonded together. On collapse, the ADP and lyso-PA products form and are released returning the enzyme to its original state. Residues that interact with ACP and MAG1 in the asBC site of the ternary complex are shown with hydrogen bond and ionic interactions indicated by hashed bonds. Hydrophobic interaction is highlighted as a black semicircle. Interactions that are likely and possible, but that are not supported by structure data, are indicated by dashed bonds. Structurally equivalent residues in cAMP-dependent protein kinase A are shown boxed with residue numbers as in PDB ID: 1ATP. (**b**) Kinase activity of DgkA as affected by site specific mutations. Kinase activity is expressed as a percentage of WT activity. Actual values are recorded in Supplementary Table 1. The one letter amino acid code is used. Residual activities of 0%, 0–0.5% and 0.5–5% are indicated by #, ** and *, respectively.

The ternary complex site, asBC, contains zinc-ACP and two lipid substrates (Fig. 1a). The zinc-ACP is close to fully extended and is secured by electrostatic, hydrogen bonding and hydrophobic interactions along its length on the relatively flat, cytosol-exposed surface of H2_C_ and H3_C_ (Fig. 1a,c,d). The enzyme has a requirement for metal ions, known to be satisfied by zinc^16^. The two zinc atoms (Zn1, Zn2) bound to ACP were identified and positioned in the complex by anomalous scattering (Fig. 1e). They are bidentately coordinated, respectively, by the β- and γ- and the α- and γ-phosphates of ACP and fasten the polyphosphate to conserved residues Glu28_C_ and Glu76_C_ (Supplementary Table 1 and Supplementary Fig. 1b) in the active site in ways that make catalytic sense (Fig. 2a). MDS illustrate interactions between the γ-phosphate and the backbone amides of Ala29_C_ and Ala30_C_ (Supplementary Movie 1), both of which contribute to the helix dipole of H1_C_.

**Figure 3:**
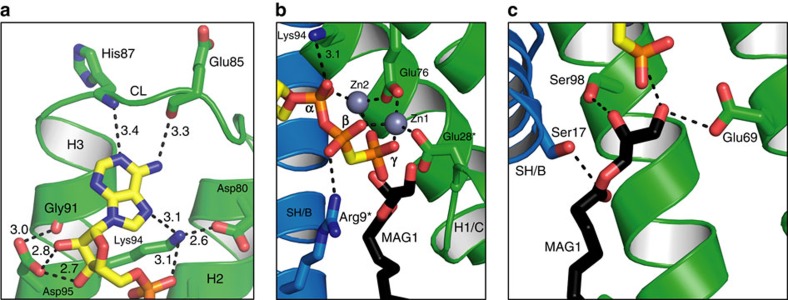
Substrate-binding sites of DgkA. (**a**) Binding of the adenosine of ACP at the extracellular surface of active site asBC. Interactions are shown as dashed lines with distances between non-hydrogen atoms in Å. Protein and ACP carbons are coloured green and yellow, respectively. Oxygen and nitrogen are coloured red and blue, respectively. (**b**) Binding of the zinc-triphosphate moiety of zinc-ACP to asBC. Lipid carbons are coloured black. Distances for zinc coordination range from 2.0 to 2.2 Å. The asterisks indicate that little or no electron density was observed for the side chains of Arg9 and Glu28. However, interactions with zinc-ACP, of the type noted, were seen with both residues in MDS (Supplementary Movie 1). (**c**) The putative active site of DgkA where the catalytic residue Glu69_C_, ACP and lipid substrate MAG1 meet in asBC. The glycerol headgroup of MAG1 could not be oriented unambiguously in the active site based on the available electron density maps, even at 2.05 Å resolution (Supplementary Fig. 4a–d). Accordingly, the interactions between MAG1 and the enzyme are shown as dashed lines only and without specifying distances. The orientation shown, with the 1-OH in hydrogen-bonding distance to the carboxyl group of Glu69, is plausible in light of a careful consideration of the available maps and the nature of the lipid substrate and the reaction being catalysed.

The two hydroxyls of ribose in ACP are tightly hydrogen bonded to the side-chain carboxyl of the essential Asp95_C_ (Fig. 3a). These contribute to substrate binding and specificity, and explain why 2′-deoxy-ATP, with one less hydroxyl anchor to the protein, is a considerably poorer substrate^2^. Hydrophobic interactions between the non-polar surface of the ribose and the methylenes of Lys94_C_, itself a critical residue^9^,^13^ (Fig. 2b), contribute to fixing the ribose in place and to having it correctly oriented (Fig. 3a).

**Figure 4:**
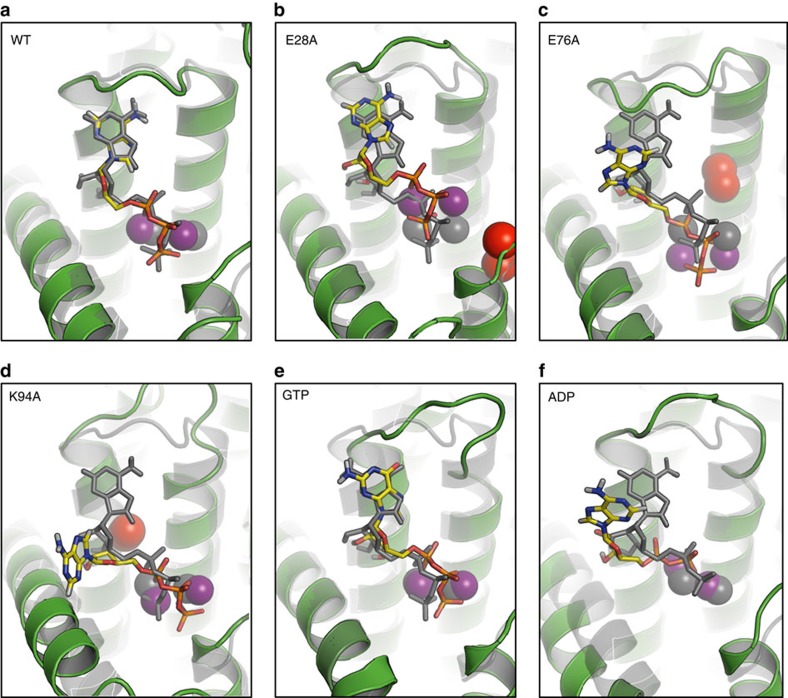
Changes in the active site of the DgkA ternary complex at the beginning and end of a 100-ns MDS. (**a**) WT simulation of ATP based on the ACP coordinates. There are limited differences in the binding site between the start (grey) and end (colour) of the MDS. (**b**) E28A mutation (red). This principally affects the binding of the Zn2 ion. In the absence of the E28 side chain the zinc ions become purely coordinated by E76 and the ATP phosphates. As a result, the entire zinc-ATP complex moves away from the protein, towards the cytoplasm. This, in turn, slightly alters the conformation of the CL. (**c**) E76A (red). To compensate for the loss of E76, the zinc ions move towards the membrane to interact with E69. This pulls the ATP in the same direction and, in turn, the CL is affected. (**d**) K94A (red). The WT residue coordinates both α-phosphate and N7 of the adenine ring of ATP. The loss of the basic side-chain releases the adenine of ATP and the binding is lost. In WT simulations, K94 forms a salt bridge with D80 and it is expected that the loss of this bridge in the D80A mutant can also explain the loss of catalytic ability in this mutant. (**e**) GTP. The major difference in dynamics is observed in the CL, where the loss of the N6 hydrogen bond with the backbone of E85 destabilizes purine binding and the CL. (**f**) ADP. The ADP molecule is expected to leave the binding site after catalysis has taken place. A change in zinc ion coordination takes place as Zn1 now coordinates the α- and β-phosphates of ADP. This relocates ADP closer to K94, which also coordinates both phosphates. In turn, K94 no longer interacts with the N7 position of the purine ring, which changes conformation, priming the ADP molecule for exit. Throughout this legend, WT refers to Δ4-DgkA (ref. 12).

The purine ring of ACP is locked in position by hydrogen bonds between the N1 and N6 of adenine and the backbone linkage of His87_C_ and Glu85_C_, respectively, in CL_C_ (Fig. 3a). Such backbone-adenine interactions form the basis of nucleotide recognition by analogy with base pairing in nucleic acids. Backbone interactions are relatively residue non-specific. This explains mutagenesis work where neither residue was identified as essential (Fig. 2b and Supplementary Table 1). Between Glu85 and His87 is Tyr86_C_ whose tyrosyl ring hovers close to the adenyl of ACP poised for π–π stacking to lock adenine firmly against the protein (Supplementary Fig. 3c,f). We refer to these three sequential residues as the clamping triad. The purine is secured too by its N7 interaction with the ɛ-amino of Lys94_C_, which also interacts with the α-phosphate of ATP, and is tethered in place by a salt bridge with Asp80_C_ (Fig. 3a). As expected, this position is, by contrast, sensitive to mutation. Together with Glu85_C_, His87_C_ and Asp95_C_, Lys94_C_ acts like a multiply tipped pincer to lock the adenosine of ACP in a specific orientation on the protein (Fig. 3a). Measured *k*_cat_ values of DgkA for guanosine triphosphate (GTP) and inosine triphosphate (ITP) are reduced by orders of magnitude compared with ATP (ref. 2). This purine selectivity makes sense in light of the ternary structure. Thus, replacing the N6 in adenine with an O6 in guanine or inosine would lead to unfavourable interactions between the two purines and adjacent backbone carbonyls of Gly83, Ser84, Glu85 and Val79. Clear evidence of instability with GTP was observed by MDS. Likewise, the crystal structure is not compatible with tight pyrimidine nucleotide binding, consistent with the observation that while adenine nucleotides dissolved crystals of DgkA, cytidine triphosphate (CTP), uridine triphosphate (UTP) and thymidine triphosphate (TTP) did not^12^. MDS show that ATP is extremely stably bound with the same orientation and interactions as ACP in the crystal structure (Fig. 4a and Supplementary Movie 1). ACP is therefore a reliable ATP analogue.

The ternary complex asBC contains two lipid substrates (MAG1, MAG2; Fig. 1a). MAG1 has been modelled into the electron density map with its headgroup deep in the protein positioned at the level of the membrane interface with its reactive 1-OH next to the γ-phosphate of ACP (Fig. 1c,f). MAG2 is in the putative lipid substrate-binding pocket, with its headgroup ∼4 Å from MAG1. Their acyl chains extend into the membrane along the hydrophobic surface of the protein. Since DAG, with two acyl chains, is the canonical lipid substrate of DgkA (ref. 2), finding a pair of MAGs as noted, implies they demark the lipid substrate-binding site.

The electron density for MAGs in the complex is variable and in parts, discontinuous (Supplementary Fig. 4a–d). However, in our studies of DgkA to date, we have crystal structures that include a total of 10 trimers with density in the active site for bound lipid that is variable (refs 12, 17 and this work). Mapping these onto a single active site makes a convincing case that lipids reside in the binding pocket (Supplementary Fig. 4e–g). Their variable definition likely reflects acyl chain flexibility and lipid movement during reaction.

The two other active sites in the complex lack nucleotide (Fig. 1b). asAB has two MAGs situated similar to those in asBC. Like asBC, asAB includes an almost complete SH that creates a well-defined active site. By contrast, the SH of asCA is not visible in density until Ser17_A_. Accordingly, this active site appears wide open (Fig. 1b). It contains just one, distant MAG.

### Reaction mechanism

Mutational studies have identified residues in DgkA that impact on catalysis (refs 9, 13 and this work). They include Thr8, Arg9, Ser17, Gly20, Glu28, Ala30, Glu34, Glu69, Asn72, Ser73, Glu76, Asp80, Gly83, Ser90, Lys94, Asp95, Gly97 and Ala100 (Fig. 2b). Convincingly, 14 out of the 18 are proximal (≤5 Å) to the nucleotide and lipid substrates in the ternary complex and all are exposed to solvent during the MDS (Fig. 3a–c, Supplementary Fig. 5). They demark the substrate binding and catalytic sites. The putative catalytic site resides on the protein at the membrane/cytosol interface where the reactive moieties of the two substrates, with disparate polarities, come together for reaction.

**Figure 5:**
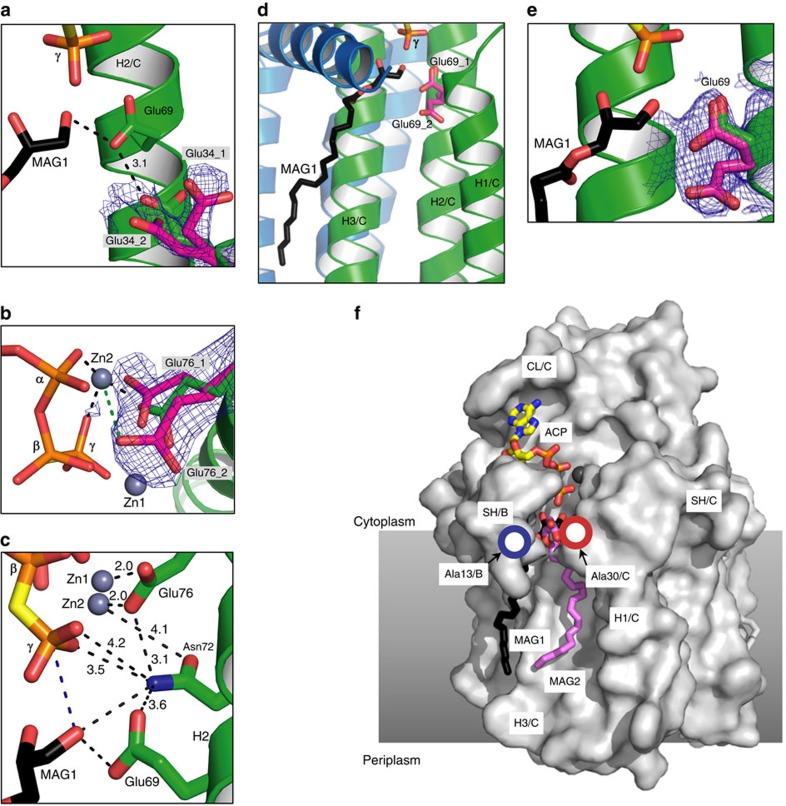
Rationalizing functional roles of highly conserved residues in DgkA not directly involved in catalysis. (**a**) The anionic carboxyl of Glu34_C_ is proposed to elevate the p*K*a of catalytic residue Glu69_C_ making it a stronger base for proton abstraction from the lipid (green protein carbons). Electron density (mesh) for alternative Glu34 conformers was observed in the XFEL data recorded at RT (purple carbons) that may enable a switch in p*K*a at Glu69. (**b**) Alternative conformations for Glu76_C_ in the XFEL structure. The first (Glu76_1) is in position to coordinate with Zn2. Black dashed lines correspond to distances 2.0–2.2 Å. The second (Glu76_2) has the coordinating oxygen at some distance (3.8 Å, green dashed line) from Zn2 where it may facilitate product release. The polyphosphate and zinc are superimposed from the ternary complex on the XFEL apo-structure. (**c**) Side-chain amide nitrogen of Asn72_C_ coordinates with carboxyls of Glu69_C_ and Glu76_C_, both essential residues. By weakly interacting with γ-phosphate oxygens, the electrophilicity of the γ-phosphorus is elevated making it more reactive. Additionally, the amide may stabilize a transient bisubstrate by being positioned close to where the pentavalent intermediate is likely to form between the 1-OH of MAG1 and the γ-phosphate of ACP (dotted blue line). Likely interactions between MAG1 and the enzyme are indicated by dashed lines only. (**d**) Alternative conformations for Glu69 observed in the XFEL structure (magenta carbons) superimposed on the DgkA ternary structure (green carbons). The first conformation (Glu69_1) is like that seen in the ternary complex and interacts with MAG1. The second (Glu69_2) extends into the membrane and may guide lipid substrate to the active site. (**e**) An expanded view of Glu69 in **d** with electron density corresponding to alternative conformers seen in the XFEL structure superposed. (**f**) The methyl side chain of Ala30_C_ (red circle) is proposed to provide room for lipid substrate and product to pass between the start of SH_B_ and the top of H1_C_ that define the gateway into and out of the active site. The Cα–Cα distance from Ala30_C_ (red circle) to Ala13_B_ (blue circle) in asBC is 10.0 Å.

The active site includes glutamates and an aspartate predicted to play roles in substrate binding and catalysis (Fig. 3a,b). Concerns have been raised generally regarding possible X-ray damage to anionic residues^18^ and to irrelevant conformational substates trapped as a result of collecting diffraction data with an intense synchrotron microbeam at 100 K (refs 19, 20). To address these issues we have obtained a structure by serial femtosecond crystallography (SFX) with an X-ray free-electron laser (XFEL) at room temperature (RT) and ahead of radiation damage^21^,^22^,^23^ (Table 1). It suggests that damage and ‘frozen' conformational substates were not major issues under current conditions and that the ternary complex structure reported here is reliable. Alternative conformers for side chains of three critical residues, Glu34, Glu69 and Glu76, were observed in the XFEL structure at RT that may be functionally relevant, as discussed (Fig. 5a,b,d,e).

Convincing kinetic and biochemical evidence supports a direct, in-line phosphoryl transfer mechanism for DgkA (ref. 2). This is in contrast to one that requires the formation of an enzyme-phosphate intermediate with subsequent phosphotransfer to the lipid substrate. The direct route displays a hallmark, trigonal bipyramidal phosphorus intermediate and a random kinetic pathway where one substrate does not exclude the binding of the other. Further, the γ-phosphate of ATP should be proximal to the 1-OH of the lipid substrate. This is what was observed in the ternary complex where the two entities are poised for reaction ∼4 Å apart (Fig. 3c). The separation is reminiscent of what has been interpreted for DgkA and for water-soluble kinases, where a tetraphosphate analogue of the bisubstrate (ATP-DAG in the case of DgkA) is a better inhibitor than the bisubstrate itself^2^. Presumably, the extra space accommodates both substrates, the reactive moieties of which are brought together for reaction by a small conformational change in the protein.

Glu69 is a conserved, essential residue in DgkA (refs 9, 13; Fig. 2b, Supplementary Table 1, Supplementary Fig. 1b). In the liganded asBC, its carboxyl is in hydrogen-bonding distance to the 1-OH of MAG1 (Fig. 3c). By analogy with other kinases^24^, we propose that Glu69 initiates catalysis by abstracting a proton from this primary hydroxyl. In this configuration, the proximal Glu34_C_ (Fig. 5a, Supplementary Fig. 6, and Supplementary Fig. 7) is predicted to have a p*K*_a_ of 7.53 and therefore will be protonated at neutral pH, acting as a hydrogen bond donor to Glu69_C_ at rest. This is a stable configuration in the MDS. Due to the hydrophobic nature of the pocket, deprotonation of Glu34_C_ will elevate the p*K*_a_ of Glu69_C_ making it a stronger base and a better general-base catalyst. We hypothesize, therefore, that deprotonation of Glu34_C_ is the trigger for catalysis in a substrate-loaded enzyme. Interestingly, in the XFEL structure of the apo-enzyme there are two conformations for Glu34 and Glu69. One of each conformer has a matching partner in the cryo structure described above that can be isolated from the MDS by switching the protonation states of Glu34_C_ and Glu69_C_. In the case of Glu69, the alternate conformation extends deeper into the membrane and has a predicted p*K*_a_ value of 8.82. This is reflected in the MDS, where protonation of Glu69_C_ induces a side-chain switch to this conformation and inversion of the hydrogen-bonding configuration with Glu34_C_. This is likely to be the side-chain rotamer after the proton has been removed from the primary hydroxyl of the lipid substrate. Alternatively, this may guide substrate lipid entering from the bilayer to the active site (Fig. 5d,e). With Glu34, the two conformers may also provide a switch that controls the p*K*_a_ and basicity of Glu69. The lipid alkoxide formed on proton abstraction is in position to attack the γ-phosphate of ATP which leads to the formation of the pentavalent intermediate, stabilized by Asn72_C_ and/or Arg9_B_ (Figs 2a, 3b and 5c). Collapse of the intermediate severs the β−γ linkage, generating lyso-phosphatidic acid and ADP as products. Since both are negatively charged, product egress might be aided by electrostatic repulsion from anionic residues Glu69_C_ and Glu76_C_ in the active site, accompanied by metal-binding site rearrangement, and by cationic residues Arg9 and Lys12 in the SH proposed to draw the lipid out as the SH flexes away from the site (Fig. 4f). On release, the active site is reset for another round of catalysis, with Glu34 reprotonating from Glu69 (Supplementary Figs 6,7).

MDS show clearly that ADP is positionally unstable in the binding site (Fig. 4f, Supplementary Movie 1). DFT simulations on MDS-sampled configurations displaying intimate MAG1 contacts (similar to Fig. 3c) suggest that 1-OH proton abstraction by Glu69_C_ is kinetically more accessible than phosphate cleavage (Supplementary Fig. 8, Methods section). In accord with the proposed pentavalent stabilization by Asn72_C_ and/or Arg9_B_ (Fig. 2a), the cleavage step is likely to be rate-limiting.

Two MAGs have been modelled in the liganded asBC. The 1-OH of MAG1 is proximal to the carboxyl of the putative catalytic Glu69_C_ (Fig. 3c). Ser98_C_ interacts with the 2-OH of MAG1 which, in the case of the canonical substrate DAG, would be in ester linkage to an acyl chain. The carbonyl oxygen at the ester linkage in MAG1 is within hydrogen-bonding distance of critically important Ser17_B_. Together, these interactions with highly conserved and/or critical residues fix the headgroup of the lipid substrate in position for proton abstraction by Glu69_C_ and for reaction with the γ-phosphorus of ATP (Fig. 3c). The lipid acyl chains reside within or close to a three-walled hydrophobic pocket created by the transmembrane stretches of H1-3_C_ (Supplementary Fig. 4e–g). The pocket appears to use the bulky and highly conserved Trp112_C_ (Supplementary Table 1 and Supplementary Fig. 1b) with the plane of its indole ring oriented, by hydrogen bonding to Ser61_C_ in the trimer core, to create a base for the pocket below which the chains do not extend (Supplementary Fig. 4f). Its location towards the bilayer mid-plane may contribute to defining lipid substrate chain-length preference.

### Comparison with other kinases

In support of the proposed mechanistic model, we note the striking resemblance between critical residues in the active site of DgkA and their equivalents in cAMP-dependent protein kinase (PKA) for which a similar catalytic mechanism is well established^24^,^25^ (Fig. 2a). To begin with, the catalytic residue in both kinases is anionic, Glu69 in DgkA and Asp166 in PKA. While magnesium is a common ATP counter-ion in kinases, zinc can serve this role in DgkA (ref. 16). Both zinc ions in the ternary complex bind electrostatically with the γ-phosphate of ACP and, by electron withdrawing effects, render the phosphorus more susceptible to attack by the lipid nucleophile. The same kind of interaction is seen in PKA. Lys94_C_ coordinates with the α-phosphate of ACP in a manner analogous to its counterpart, Lys72, in PKA. Lys94_C_ is stabilized in position by a critical Asp80_C_, just like the Lys72-Glu91 pair in PKA. The essential Glu76_C_ chelates the two zincs which, in turn, bind the triphosphate of ACP. The equivalent residue in PKA is Asp184. Parenthetically, a second conformation for the side chain of Glu76_C_ is present in the XFEL apo-structure which is more removed from the metal ions; it may play a role in product release (Fig. 5b). Arg9 is functionally important and mutating it dramatically alters DgkA activity. While its side chain lacks full density in the DgkA complex, one of its possible conformers makes a convincing interaction with the α-phosphate of ACP, reminiscent of the role played by Lys168 in PKA. Asn72 is essential in DgkA (Fig. 2b and Supplementary Table 1). Its side-chain carbonyl is 4.1 Å from Zn2 while its amide nitrogen bridges the essential Glu69 and Glu76 (Fig. 5c). The equivalent residue in PKA is Asn171; it bridges Asp166 and Asp184 and its carbonyl is 2.1 Å from manganese (Mn1).

**Figure 6:**
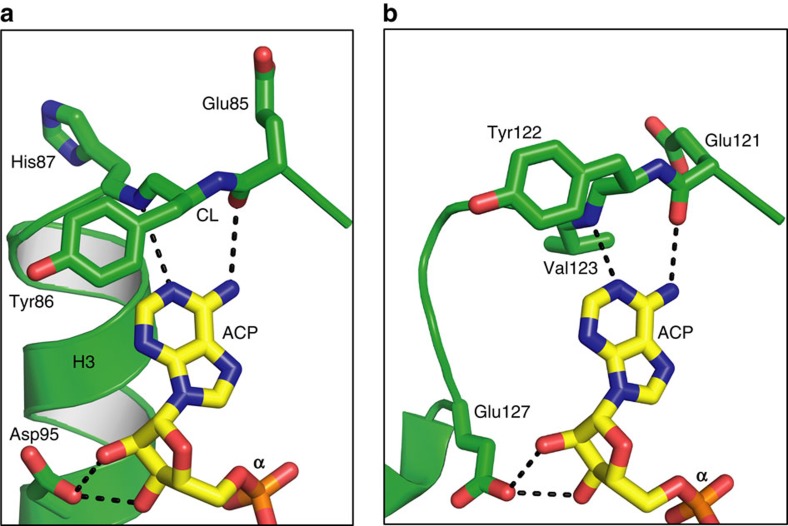
Similarities in the binding coordination of adenosine by DgkA and cAMP-dependent protein kinase A. (**a**) DgkA, this study. (**b**) Protein kinase A, PDB ID: 1ATP. Only the similarities are highlighted. In **a** one surface of the adenosine moiety faces the protein, the other faces the cytoplasm. In **b** the adenosine is sandwiched between the N- and C-terminal lobes of PKA.

The similarity between the binding sites of DgkA and PKA extends to the adenosine end of the nucleotide. Thus, the aforementioned clamping triad on adenine in the CL of DgkA that includes Glu85-Tyr86-His87 is matched by an almost identical triad in PKA represented by Glu121-Tyr122-Val123 (Figs 2a and 6). At the same time, the two ribose hydroxyls are anchored by Asp95 in DgkA and by Glu127 in PKA. The resemblance between residues in and chemical architecture of the active sites of DgkA and PKA is remarkable given that the two kinases are evolutionarily unrelated and have essentially no primary through quaternary structure similarities.

### Mutagenesis

With the ternary complex structure in hand, hypotheses regarding the role of specific residues in DgkA enzymatic activity were tested by systematic mutagenesis. Kinetic measurements were performed with the mutants reconstituted in the LCP. A total of 23 sites were mutated with the types screened dictated by the putative role for the particular residue. The results are summarized in Fig. 2b and Supplementary Table 1 along with data from separate mutational studies^9^,^13^. A few notable mutants will be discussed here, as follows. (i) Glu69 is the putative catalytic residue in DgkA (Figs 2a and 3c), in contrast to Asp which serves that function in serine protein kinases. Strikingly, Glu69 tolerated none of the mutations tested, one of which was Asp (p*K*_a_ 3.8). This could be a spatial effect because the side chain of Asp is too short. It may also have to do with the fact that the 1-OH in glycerides like MAG and DAG have p*K*a values (13.6) higher than those of PKA substrates Ser/Thr (p*K*_a_ 13.0). Glycerides therefore may require the carboxyl of Glu, with a p*K*_a_ of 4.3, or higher enabled by nearby Glu34, for proton abstraction. (ii) The conserved Asp95 coordinates the hydroxyls of ribose in ACP, preserved throughout the MDS, and the carbonyl of Gly91 (Fig. 3a). Mutating it to Ala essentially abolished activity. However, Glu with its longer side chain did substitute but activity was reduced by 76%. By contrast, Asn had a minor effect which makes sense given that its side-chain amide can hydrogen bond with carbonyls and hydroxyls. (iii) Ala30 resides on H1 in the active site entrance at the membrane interface (Fig. 5f). Its methyl side chain was speculated to enable lipids with small headgroups to enter the site. Mutating it to the bulky leucine reduced activity by 93% consistent with a putative gatekeeping role. (iv) Gly83 in the CL was proposed to facilitate the helix-coil transition enabling adenine binding (Supplementary Fig. 3). Mutating it to Pro was expected to compromise such activity. Indeed, the Gly83Pro mutant was virtually dead enzymatically; MDS show dynamic instability of the CL (Supplementary Movie 2) and (v) finally, Asn72 plays a key role in catalysis. Its side-chain amide bridges Glu69 and Glu76 (Fig. 5c) both of which are essential. These H-bonds bridge the two essential residues throughout the MDS. The Asn72Ala mutant is catalytically inactive. For all intents and purposes, so too are the Asn72Gln and Asn72Asp mutants. These data show how important both the precise chemistry and the architecture of this part of the binding/active site are for catalysis. The structure-based, hypothesis-testing mutations and others in Fig. 2b and Supplementary Table 1 lend credence to the complex model and mechanism of kinase action.

## Discussion

DgkA is the tiniest of kinases. With just 121 residues and a trimeric constitution, three kinase catalytic sites have been fashioned (Fig. 1). Each acts at the membrane/cytosol interface. The rest of the protein creates binding pockets on either side that position the reactive ends of two large substrate molecules with dramatically different polarities in juxtaposition for reaction. The ternary structure explains in atomic detail how the nucleotide and lipid-binding sites interact with substrate molecules. The many, contact site-docking defines a specific orientation for the nucleotide that presumably is optimized for transphosphorylation. Particularly striking is the similarity between the binding site architecture of this bacterial DgkA and mammalian PKA (Figs 2a and 6), kinases that bear no resemblance at any other level of structure. Thus, these defined points of chemical constitution arrayed precisely in three-dimensional space have evolved independently achieving a similar end in entirely different protein contexts – a convincing case of convergent evolution.

DgkA's small size shows that a functioning kinase can be built with very little protein mass. The ternary structure points to a minimum required for that purpose. Inspecting the complex suggests that a pair of helices, each 3-4 turns long, connected by a 7-residue loop (Supplementary Fig. 3) might be all that is needed to create an ATP-binding module. The ternary structure should serve as a template for rational protein and enzyme design, optimizable for solubility, stability, specificity and catalytic efficiency.

Kinetic studies suggest that DgkA functions with a moderate level of heteroallostery^1^. Thus, the binding of one substrate influences (in this case positively) that of its co-substrate. In a multimer like DgkA, it is possible for heteroallostery to come about through inter-subunit signalling. The fact that any given active site is a composite, with components from at least two subunits, suggests a way for the substrate-bound status of one site to be relayed to another; the SH is an obvious means for inter-subunit information transmission. In this way, the enzyme could present simultaneously three active sites in distinct states. We find some evidence for this in the ternary structure where only one of the three active sites is occupied by ACP. Further, two of the sites in the CL region, which clamps onto the nucleotide base, are profoundly different (Supplementary Fig. 3). Neither CL is involved in crystal contacts. Presumably therefore both are physiologically relevant. We propose that the three sites in DgkA are in different states at any given time during catalysis. However, it is possible to model a symmetrical DgkA trimer that is stable in MDS, indicating that the enzyme may adopt a symmetrical configuration. This does not imply that all three sites are catalytically active at the same time. Additional mechanistic and structure work is required to evaluate allosteric coupling within the enzyme.

As a trimer situated with half its bulk in the membrane and half in the cytosol, DgkA has features that make it a good biocatalyst. Its three active sites are arranged around the enzyme's periphery (Fig. 1b) opening to the cytosol and the membrane for easy access and egress of substrates and products. This naturally raises the probability of productive encounters between substrates and binding sites, thus elevating the reaction rate compared to a hypothetical solitary active site. Conformational changes, where needed for turnover, would appear to be relatively minor involving small segments such as the CL for nucleotide and the SH for lipid and nucleotide binding. This should ensure efficient substrate loading and product release. The reduction in dimensionality, as a result of lipid substrate and enzyme confinement to a two-dimensional membrane, increases overall efficiency of delivery. The amphiphilic nature of the lipid and of the membrane in which it and the kinase reside, ensure the spontaneous and proper alignment of substrate in the lipid-binding site for reaction. These features of trimeric DgkA undoubtedly contribute to making it an efficient biocatalyst, described previously as an evolutionarily optimized integral membrane enzyme.

## Methods

### Molecular biology and protein production

All but two of the DgkA mutants were generated by PCR-based site-directed mutagenesis using the plasmid pTrcHisB-DgkA-WT (ref. 14) as the template. In the case of E34D and E34A, constructs with the desired mutation could not be produced after several rounds of mutagenesis trials. Instead, the corresponding genes were synthesized chemically (Genescript). All mutations were confirmed by DNA sequencing (MWG Biotech). Detailed purification procedures are described in ref. 14. Briefly, *E. coli* WH1061 cells carrying DgkA plasmids and grown in Luria-Bertani broth were induced at an optical density of 0.6 for 3 h at 37  °C with 1 mM IPTG. All subsequent steps were carried out at 4 °C unless otherwise specified. For crystallization, cells from 1 l of culture were lyased by sonication in buffer containing 0.2 mM TCEP, 0.3 M NaCl, 0.2 mg ml^−1^ lysozyme, 50 μg ml^−1^ DNAase, 10 μM BHT, 1 mM PMSF, 0.2 mM EDTA, 5 mM MgCl_2_ and 75 mM Tris/HCl pH 7.8. Empigen BB was added to the cell lysate at 3.3% (w/v) to solubilize DgkA for 1 h. Unsolublized material was removed by centrifugation at 9,000*g* for 10 min. The supernatant containing N-terminally His-tagged DgkA was allowed to bind to 4 ml of Ni-NTA resin for 1 h. The resin was packed in a gravity column, which was washed with 3% (w/v) Empigen BB, 10 mM imidazole in Buffer A (0.3 M NaCl, 0.2 mM TCEP, 40 mM HEPES pH 7.5) and 1.5% (w/v) Empigen BB, 40 mM imidazole in Buffer A. The detergent Empigen was then exchanged to *n*-decyl-β-D-maltopyranoside (DM) by washing the column with 12 column volumes of buffer containing 0.25% (w/v) DM, 50 mM LiCl, 0.2 mM TCEP, 20 mM HEPES pH 7.5. Protein was eluted with 0.25 M imidazole in buffer containing 0.5% (w/v) DM, 1 mM TECP, 50 mM LiCl and 10 mM HEPES pH 7.5. Pooled fractions were concentrated and run through a size-exclusion chromatography with a Superdex 200 16/600 column in buffer containing 0.25% (w/v) DM, 1 mM TCEP, 0.1 M NaCl and 10 mM Tris HCl pH 7.5. Fractions corresponding to the central peak were pooled. Protein was concentrated to 12 mg ml^−1^, flashed frozen in liquid nitrogen as 11 μl aliquots and stored at −80 °C until use. For functional assays, protein was purified from 0.1 l of cell culture, omitting the size-exclusion chromatography step. Size-exclusion chromatography had no effect on specific activity.

### Enzyme assays

*In meso* measurements of WT and mutant DgkA kinase activity were carried out as follows^15^. DgkA was reconstituted into LCP by mixing the protein solution with a 1.5-fold volume of monoolein. Five microliters of LCP was dispensed into each well of a 96-well plate. The coupled enzyme assay reaction was initiated by adding 200 μl of Assay Mix (20 mM ATP, 0.1 mM EDTA, 0.1 mM EGTA, 55 mM magnesium acetate, 1 mM phosphoenolpyruvic acid, 0.2 mM dithiothreitol, 50 mM LiCl, 0.4 mM NADH, 20 U ml^−1^ of pyruvate kinase and lactate dehydrogenase, and 75 mM PIPES pH 6.9) pre-warmed to 30 °C. The decrease in A_340_, caused by the oxidation of NADH which is coupled to ATP–ADP conversion, was recorded every 6–10 s over a period of 1 h. For WT enzyme and fully functional constructs, the protein concentration used for reconstitution into the mesophase was 0.066 mg ml^−1^. For mutants with <∼70% of WT activity, protein concentration was adjusted to 0.1−5 mg ml^−1^ to increase measurement sensitivity.

### Crystallization

*In meso* crystallization of the apo form of Δ4-DgkA for synchrotron radiation data collection was carried out using an *in meso* robot^15^. The 96-well glass sandwich crystallization plates were incubated at 4 °C for crystal growth^15^. The precipitant solution contained 7–9% (v/v) 2-methyl-2,4-pentanediol (MPD), 0.1 M NaCl, 0.1 M KNO_3_, 0.1 M Na_3_C_6_H_5_O_7_ (sodium citrate) pH 5.6 for 9.9 MAG, and 4—7% (v/v) MPD, 0.1 M NaCl, 0.06 M Mg(CH_3_COO)_2_ (magnesium acetate), 0.05 M Na_3_C_6_H_5_O_7_ for 7.9 MAG. Crystals were harvested and snap cooled in liquid nitrogen^26^.

To produce crystals of DgkA in complex with an ATP analogue, extensive screening was performed that involved exploring protein constructs, crystal forms, host MAGs, analogues, crystallization precipitants and temperature. Trials with Δ4-DgkA in 9.9 MAG with zinc-ACP at 4 °C eventually provided crystals and a structure at 2.70 Å resolution. Crystals were soaked directly in glass sandwich plates for 2 h at 4 °C using precipitant solution (7–10% (v/v) MPD, 0.1 M NaCl, 0.1 M KNO_3_, 0.1 M Na_3_C_6_H_5_O_7_ pH 5.6) supplemented with 60 mM Zn(CH_3_COO)_2_ and 10 mM ACP. Harvesting was performed, as noted above.

For measurements with the XFEL, crystallization was carried out at 20 °C in coupled micro-syringes using 7.9 MAG as host lipid^27^,^28^,^29^. The precipitant solution contained 0.2% (v/v) MPD, 0.1 M NaCl, 0.05 M Na_3_C_6_H_5_O_7_ pH 5.6. Microcrystals were identified by bright-field and polarized light microscopy, SONICC and tryptophan fluorescence at the Linac Coherent Light Source (LCLS). Crystal-laden mesophase was transferred to an LCP injector and extruded for data collection into an evacuated chamber at 20 °C, as described below.

### Synchrotron data collection and processing

Synchrotron diffraction data were collected at beamline 23-ID-B, Advanced Photon Source (USA), I24, Diamond Light Source (UK) and PXII, Swiss Light Source (SLS, Switzerland) at 100 K. At the Advanced Photon Source, data were collected with a 1° oscillation and a 1-s exposure per frame, a collimated beam size of 10 μm, and a sample-to-detector distance of 350-500 mm, with a MAR 300 CCD detector using 1.033 Å X-rays. At the Diamond Light Source, data were collected with a 0.2° oscillation and a 0.2-s exposure per frame, a microfocus beam size of 10 μm and a sample-to-detector distance of 500–650 mm, with a Pilatus 6M detector using 0.978 Å wavelength X-rays. At the SLS, data were collected with a 0.1 oscillation and 0.1-s exposure per frame, a collimated beam size of 10 × 15 μm^2^ and a sample-to-detector distance of 450–600 mm, with a Pilatus 6M detector using 1.033 Å X-rays.

Synchrotron-derived diffraction data were reduced with xia2 (ref. 30) using XDS (ref. 31) and XSCALE (Table 1). Optimum data wedges were identified by data quality and isomorphous unit cell parameters. Complete data sets for Δ4-99MAG-ACP and Δ7-79MAG (Table 1) were obtained by merging data collected from 4 and 6 crystals, respectively. A single crystal was used for the anomalous data collected at the zinc edge (Zn-edge Δ4-99MAG-ACP in Table 1).

### XFEL data collection and processing

XFEL data were collected using the CXI instrument^32^ at the LCLS at SLAC National Accelerator Laboratory^27^,^33^. The LCLS was operated at a wavelength of 1.302 Å (9.5 keV) delivering X-ray pulses of 50 fs pulse duration and with 8 × 10^11^ photons per pulse focused into a spot size of ∼1.5 μm × 1.5 μm^2^ using a pair of Kirkpatrick–Baez mirrors. Needle-shaped protein microcrystals ranging in length from 10 to 40 μm dispersed in the LCP were injected into the focus region at 0.17 μl min^−1^ using a continuously flowing LCP injector with a nozzle capillary diameter of 50 μm (ref. 27). Single-shot diffraction patterns of randomly oriented crystals were recorded at a rate of 7,200 patterns per minute (120 Hz) using the Cornell-SLAC hybrid Pixel Array Detector (CSPAD) detector^34^. The sample-to-detector distance of ∼122 mm corresponded to a maximum obtainable resolution of 2.0 Å at 1.302 Å wavelength. Data collection required 4.5 h of beamtime and used 42 μl mesophase corresponding to 220 μg protein.

A total of 1,987,632 frames were collected, of which 180,031 (9.1%) were identified as ‘hits' having crystal diffraction by the software package Cheetah^35^, from which 140,440 (78.0%) could be indexed and integrated using CrystFEL (ref. 36). The location of individual sensors on the CSPAD detector was determined to better than one pixel error by comparison of predicted and observed Bragg peak locations, and was key to obtaining a high indexing rate and data quality. Reflection intensities from all indexed diffraction patterns were integrated without applying a per-pattern resolution cut-off and merged in Point Group *mmm* using CrystFEL to produce one set of reflection intensities for structure determination. XFEL data could be evaluated to 2.15 Å, with a CC* of 0.998 (0.30 in the highest resolution shell) and I/sigma of 19.0 (0.33 in the highest resolution shell). Selection of the highest resolution shell (2.18 Å) was based on CC* and on improvements in *R*_work_/*R*_free_ values, as well as map quality on stepwise screening higher resolution limits (2.13, 2.15, 2.18, 2.2 and 2.4 Å) during refinement^37^.

### Structure solution, model building and refinement

Structures were solved by molecular replacement (MR) using published models (PDB ID: 3ZE3 and 3ZE4) with Phaser^38^. Coot^39^ and Phenix^40^ were used for manual building and refinement, respectively. Ligands were built based on feature enhanced maps^41^ and composite omit 2Fo-Fc maps. Lang *et al*.^42^ reported that the noise level in 2Fo-Fc maps is generally over estimated. Accordingly, the 2Fo-Fc map contoured at 0.7 σ, together with the strong electron density in the chain region of other DgkA structures (Supplementary Fig. 4), informed building the acyl chain of MAG1 in the asBC active site. The position of the two zinc ions were fixed during refinement based on an anomalous difference map (Fig. 1e) generated from X-ray diffraction data collected at the zinc edge (1.28238 Å, Table 1). The feature enhanced map (FEM)^41^ of the Zn-ACP moiety was calculated as follows. MR was performed with a DgkA trimer crystallized in the absence of nucleotide and an FEM was calculated directly from the MR solution using Phenix without adding nucleotide to the model. For the MAG1, the FEM was calculated as for the Zn-ACP but with MAG1 in place. Figures were prepared using PyMol.

### Molecular dynamics simulations

All MDS were performed using GROMACS v5.0 (ref. 43). Initial Coarse Grained (CG) MD simulations using the Martini 2.2 force field were run for 1 μs to permit the assembly and equilibration of a dipalmitoylphosphatidylglycerol (DPPG): dipalmitoylphosphatidylethanolamine (DPPE; 1:3 mole ratio) bilayer around DgkA (ref. 44). The end-snapshot of the CG simulations were then converted to atomic detail with the crystal structure aligned with the CG protein within the assembled lipid bilayer^45^. The systems were then equilibrated for 1 ns with the protein restrained before 100 ns of unrestrained atomistic MD using the Gromos53a6 force field^46^. In almost all cases, ATP, Zn^2+^ and MAG were included. In one instance, GTP was included instead of ATP. In a second case, ADP and lyso-phosphatidic acid were included in place of ATP and MAG. Parameters for catalytic intermediates were also designed, with the associated coordinates simulated in the complex. Systems were neutralized with a 150-mM concentration of NaCl. *In silico* mutagenesis was performed using PyMol.

### Density functional theory

Modelling chemical reactions is not possible with (classical) MDS. Accordingly, using *ab initio*-based DFT molecular-simulation techniques to probe putative reaction paths and energetics is of relevance and help to enzyme reaction mechanism studies. Gaussian 09 software B3LYP/6-31G(d,p) simulations were performed on MDS configurations like Fig. 3c, featuring intimate MAG1 contacts; other configurations were less energetically plausible. MM (AMBER), ONIOM-QM/MM (ref. 47) and DFT geometry-optimization led to retaining zinc atoms and residues within ∼7 Å (244 atoms) and ∼8 Å (365 atoms) of MAG1, for MAG1's reliable energetic and geometric interactions.

Examining carefully MDS-sampled, ‘cut-down' configurations of the ternary complex's active site similar to Fig. 3c, the energetics of possible interactions of 1-OH with neighbouring protein residues were studied. Both geometry optimization and systematic movement of the lipid substrate's 1-OH proton towards nearby residues were used to examine the change in the system's energy along this proton abstraction path. In DFT, albeit approximate, the electron probability density around nuclei is determined, so geometry optimization can ‘relax' and move the nuclear coordinates to lower the system's energy to a (local or global) minimum. Relaxation of particular configurations could include 1-OH proton transfer, or rearrangement of bonds, or the formation of a (near-) covalent bond between an 1-O oxyanion (or even 1-OH with proton intact) and the γ-phosphorus atom to form a pentavalent intermediate structure (Supplementary Fig. 6). A key point regarding these possible events is their energetic (and kinetic) ‘accessibility', especially in relation to background thermal energy ∼RT. At room temperature (RT) is around 0.6 kcal mol^−1^. Accordingly, such reaction events accessible for routine observation during geometry optimization would typically have energy barriers of ∼2–3 kcal mol^−1^, or less. Indeed, the connection between reaction-energy barriers and approximate rates is well-established in chemical kinetics. On this basis, we found that the only likely interaction with protein residues for reaction of 1-OH was with Glu69, featuring a prominent hydrogen bond with the proton in 1-OH pointing directly to Glu69. In some cases, geometry optimization entailed the direct transfer of the proton to Glu69, suggesting an energy barrier of ∼2–3 kcal mol^−1^, or less. The relative potential-energy profile for this is shown in Supplementary Fig. 8b, where the ‘kink' in slope denotes the rupture of the O–H covalent bond prior to formation of the bond with Glu69. Because movement along this direct path (cf. Supplementary Fig. 8a) does not necessarily follow a minimum-energy path across the potential-energy landscape (akin to taking an ‘as-the-crow-flies' route through a valley, rather than following a slightly meandering river), the ∼5 kcal mol^−1^ barrier is an overestimate, and direct transfer in structure relaxation suggests the actual barrier is lower (see above). The sharply ‘downhill' energetic nature of proton transfer indicates an approach towards spontaneity for this event. This transfer is facilitated greatly by the prominent hydrogen bond's strong electrostatic interaction, especially given the high charge-to-mass ratio of the proton.

Optimization of the alkoxide form led to the formation of a somewhat strained, imperfect covalent bond for the resultant (1-O) oxyanion (*sans* proton) with the γ-phosphorus atom, not unusual for a pentavalent intermediate, suggesting reasonable kinetic accessibility with a ∼2–3 kcal mol^−1^ barrier. However, further structure relaxation did not suggest any rupture (or meaningful stretch/strain) of the bridging γ-P–O bond. Extending systematically this covalent bond until breakage, the relative potential-energy profile (Supplementary Fig. 8b) suggests a reaction-energy barrier of ∼15 kcal mol^−1^. Unlike the hydrogen bond in proton transfer, there is no appreciable local electrostatic interaction in this bond. The much larger mass of P and O atoms and inertia of the MAG1 and to-be-formed ADP moieties compounds this, altering the character of this potential-energy profile. With a large positive barrier to be crossed, it is much less kinetically favourable. Here, the substantial positive-energy ‘hill' shows clearly the unfavourable nature of extending the bond from a local minimum in the immediate vicinity of the ‘reactant' state, hindered by less electrostatic interaction, until rupture beyond 0.5–0.6 Å. This is somewhat consistent with previous computational estimates of 26.7 kcal mol^−1^ (based on potential-energy differences), albeit for cleavage of ATP (only) in explicit water, and not in the current protein milieu^48^. Such ‘as-the-crow-flies' overestimates of energy barriers *vis-à-vis* relatively (but not completely) ‘straight' paths (akin to those shown in Supplementary Fig. 8a and traversed in b) is around double. This would suggest a γ-P–O cleavage energy barrier of ∼8–10 kcal mol^−1^. This substantially larger value compared with proton abstraction indicates that phosphate cleavage is rate-limiting. This is consistent with the proposed stabilization by Asn72 and/or Arg9 of the pentavalent intermediate (Fig. 2a), and MDS evidence pointing to Arg9-mediated stabilization.

## Additional information

**Accession codes**: Atomic coordinates and structure factors for Δ4-99MAG-ACP, Δ4-99MAG, Δ7-79MAG-FEL and Δ7-79MAG are deposited in the Protein Data Bank under accession codes 4UXX, 4UXW, 4UYO and 4UXZ, respectively.

**How to cite this article:** Li, D. *et al*. Ternary structure reveals mechanism of a membrane diacylglycerol kinase. *Nat. Commun.* 6:10140 doi: 10.1038/ncomms10140 (2015).

## Supplementary Material

Supplementary Information Supplementary Figures 1-8, Supplementary Table 1 and Supplementary References.

Supplementary Movie 1 DgkA MD Simulations. a, DgkA trimer in a lipid bilayer. ATP and MAG1 shown as yellow spheres. Zinc ions shown as grey spheres. Phosphate lipid head group atoms shown as orange spheres. b, Focus on the ATP binding site. ATP and residues E28, N72, E76, E85, H87, K94 and D95 shown as sticks. The stability of the dynamics highlights the high fidelity of the crystal structure coordinates. c, Simulations with ADP and LPA, shown as sticks. The Zn1 ion moves to bridge the α- and β-phosphates of ADP. This slightly repositions the ADP molecule, so that K94 interacts with both α- and β-phosphates. The relocation removes the K94 hydrogen bond with N7 and thus the adenine ring is more likely to move, breaking the hydrogen bonds with E85 and H87. Once free, the enhanced adenine dynamics ruptures the hydrogen bonds with D95, leaving only interactions between Zn1 and K94 and the two remaining phosphates. Zn2 bridges the β-phosphate of ADP and the phosphate of LPA in this state.

Supplementary Movie 2 DgkA Mutant MD Simulations. In each case, the mutated residue is identifiable by a black sphere. a, E28A. In the simulations, the zinc-ATP complex moves towards the cytoplasm, away from the membrane and the MAG1 head group. Loss of the E28 side chain is likely to affect the binding of Zn2 in the absence of ATP. b, E76A. To compensate for the loss of E76, the zinc-ATP complex moves towards the membrane, so that E69 can fulfil the role of E76 in coordinating the two zinc ions. c, G83P. The proline mutation (spheres) appears to destabilise the CL, breaking the hydrogen bonds between the adenine ring and E85 and H87. d, K94A. This mutation rapidly destabilises both the adenine ring and α-phosphate of ATP due to loss of hydrogen bonds. As a result, the head group of ATP flips out of the binding site.

## Figures and Tables

**Table 1:** Crystallization conditions, data collection and refinement statistics.

	**Δ4-99MAG-ACP**	**Δ4-99MAG-ACP-Zn**	**Δ4-99MAG**	Δ7-79MAG-FEL*	Δ7-79MAG*
Crystallization conditions					
Construct	Δ4	Δ4	Δ4	Δ7	Δ7
Host lipid	9.9 MAG	9.9 MAG	9.9 MAG	7.9 MAG	7.9 MAG
Zinc-ACP	Yes/soak	Yes/soak	No	No	No
Temperature (K)	277	277	277	293	277
					
Data collection					
X-ray source	Synchrotron	Synchrotron	Synchrotron	FEL	Synchrotron
Temperature (K)	100	100	100	294	100
Space group	P3_1_21	P3_1_21	P3_1_21	P2_1_2_1_2_1_	P2_1_2_1_2_1_
Cell dimensions					
*a*, *b*, *c* (Å)	72.94, 72.94, 195.80	73.08, 73.08, 196.00	72.80, 72.80, 199.32	75.30, 91.80, 141.70	75.02, 91.31, 143.70
*α*, β, γ (°)	90, 90, 120	90, 90, 120	90, 90, 120	90, 90, 90	90, 90, 90
Wavelength (Å)	1.0331	1.2824	1.0332	1.302	1.0332
Resolution (Å)	53.07–2.70 (2.77–2.70)†	53.17–3.20 (3.29–3.20)†	45.73–3.15 (3.23–3.15)†	40.50–2.18 (2.24–2.18)†	75.02–2.18 (2.24–2.18)†
*R*_merge_‡	0.117 (0.855)†	0.099 (0.667)†	0.126 (1.739)†	n/a	0.100 (1.267)†
*R*_pim_‡,(Syn) or *R*_split_ (FEL)‡	0.051 (0.504)†	0.058 (0.397)†	0.042 (0.573)†	0.068 (2.380)†	0.058 (0.735)†
*I*/σ*I*	11.9 (1.6)†	12.8 (2.8)†	11.9 (1.4)†	19.0 (0.44)†	12.1 (1.7)†
Completeness (%)	99.3 (94.1)†	99.4 (99.9)†	99.7 (99.8)†	100 (100)†	99.1 (96.8)†
Multiplicity	9.1 (5.3)†	7.0 (7.2)†	10.7 (11.0)†	3,466 (3,519)†	6.7 (6.4) †
CC*‡	0.999 (0.861)†	0.999 (0.971)†	0.999 (0.847)†	0.999 (0.578)†	0.999 (0.736)†
					
Refinement					
Resolution (Å)	53.08–2.70		45.73–3.15	40.01–2.18	57.97–2.18
No. reflections	17,165		11,121	51,799	51,436
*R*_work_/*R*_free_§	0.219 (0.278)/0.258 (0.340)§		0.224 (0.272)/0.268 (0.329)§	0.208 (0.378)/0.236 (0.388)§	0.200 (0.311)/0.235 (0.369)§
No. atoms	2,804		2,754	5,225	5,263
Protein	2,584		2,591	4,919	4,861
Ligand/ion	207		163	221	271
Water	13		0	85	131
					
B-factors (Å^2^)	92.9		97.8	70.4	60.1
Protein	91.3		97.2	69.5	59.0
Ligand/ion	112.7		106.7	90.0	77.4
Water	92.4		n/a	71.8	64.7
R.m.s deviations					
Bond lengths (Å)	0.012		0.016	0.008	0.014
Bond angles (°)	0.749		1.286	0.963	0.896
PDB ID	4UXX		4UXW	4UYO	4UXZ

^*^The Cα root-mean-square deviation (RMSD) between Δ7-79MAG-FEL and Δ7-79MAG is 0.35 Å over 598 residues.

^†^Highest resolution shell is shown in parenthesis.

^‡^
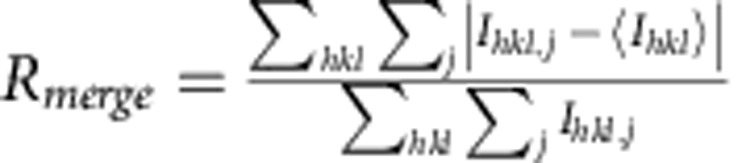
, 
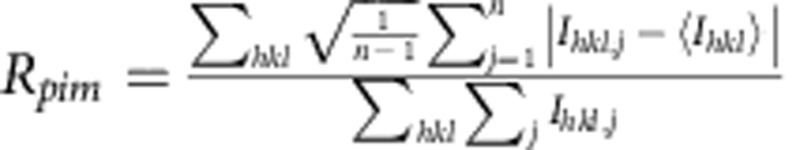
. 
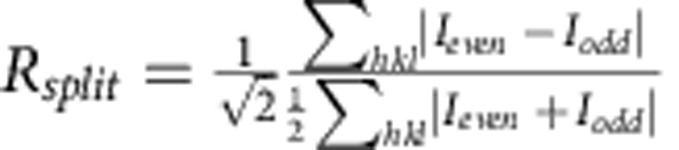
. 
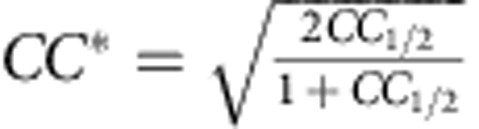

^§^R factors for the highest resolution shell are shown in parenthesis.
