# Supplementary material for: Ternary structure reveals mechanism of a membrane diacylglycerol kinase
Source: Nat Commun. 2015 Dec 17;6:10140. doi: 10.1038/ncomms10140 (PMC4703834; doi:10.1038/ncomms10140)
Supplement: Supplementary Information — Supplementary Figures 1-8, Supplementary Table 1 and Supplementary References. [file ncomms10140-s1.pdf]

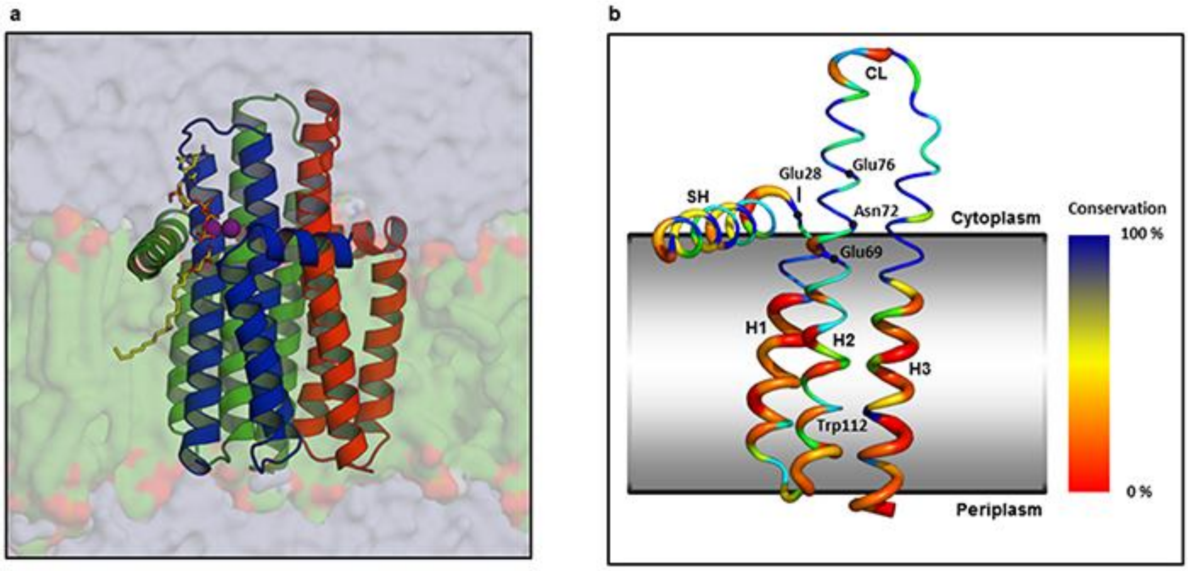

Supplementary Figure 1 | Membrane disposition of and residue conservation in DgkA. **a**, Disposition of DgkA in a membrane by MDS. DgkA ternary complex, with zinc-ACP and MAG1 (yellow sticks), embedded in a bilayer composed of DPPE and DPPG lipids (green surface). Aqueous solution is 150 mM NaCl in water (light blue surface). Both the zinc-ACP and the glycerol headgroup of MAG1 are fully solvated. The remainder of MAG1 is immersed in the membrane. The hydrophobic thickness of the DgkA trimer is ~35 Å and, to accommodate the enzyme the DPPE:DPPG bilayer thins from its bulk thickness of 40 Å. The amphiphilic SH anchors the active site at the interface while the periplasmic surface of the membrane thins locally to match the protein's hydrophobic surface. The γ-phosphate of the nucleotide and the 1-OH of the lipid substrate approach one another at the membrane/aqueous interface. **b**, Percent residue conservation is shown in putty representation with line girth and color ranging from thin and blue for 100 % to thick and red for 0 % conservation. Conservation values were calculated based on a sequence alignment of 1,000 unique sequences taken from a BLAST search on the UniRef100 database. The locations of highly conserved residues referred to in the text are indicated. See also **Supplementary Table 1**. The model used here is suB from the ternary complex.

a

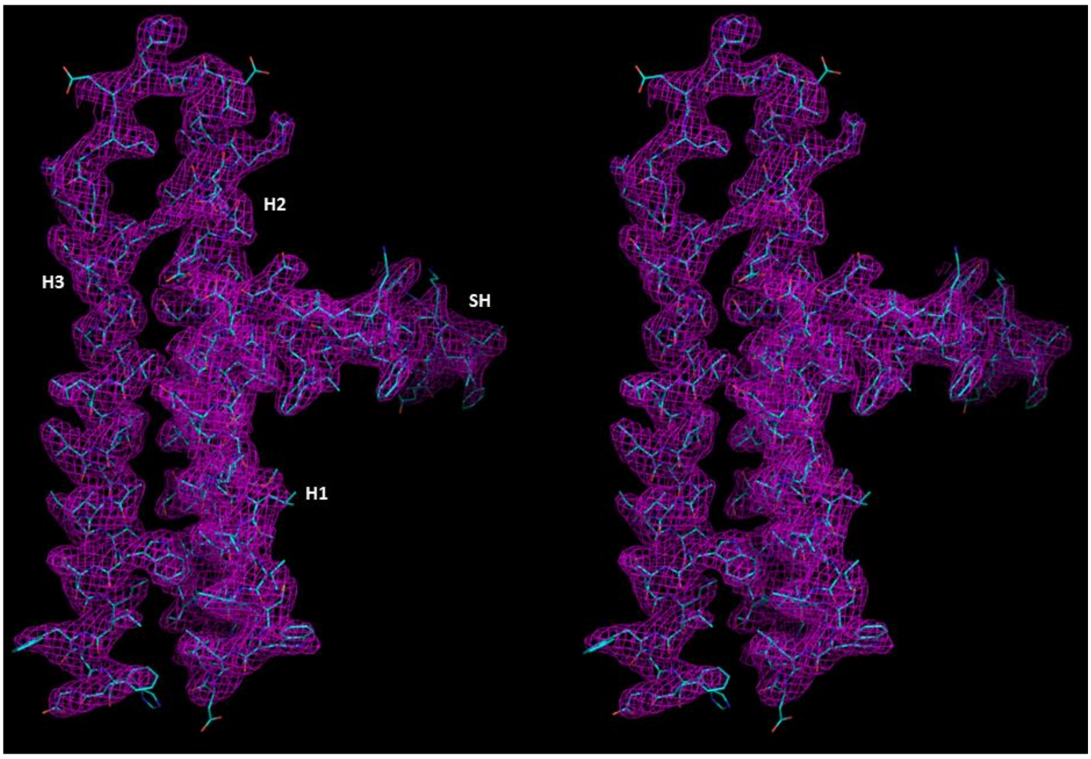

b

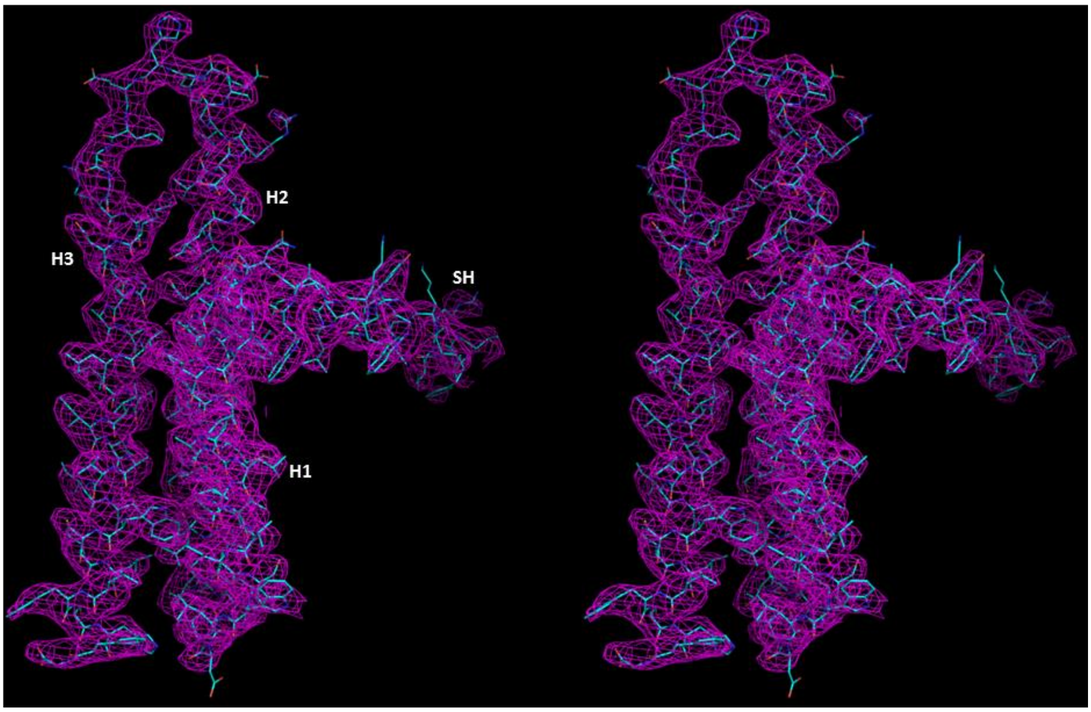

c

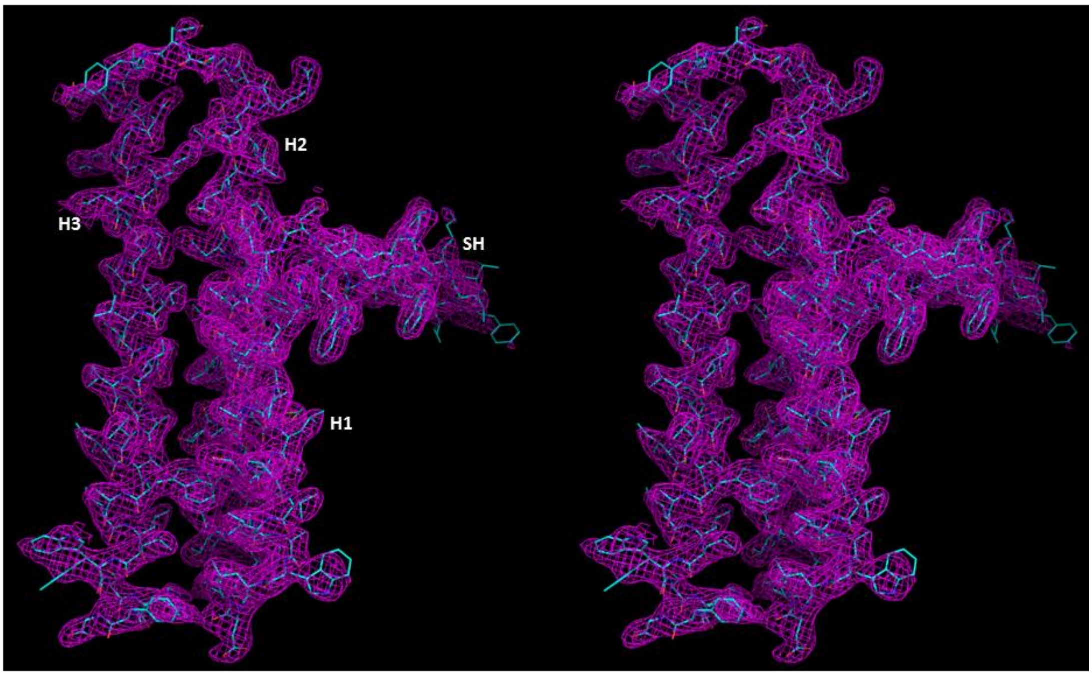

d

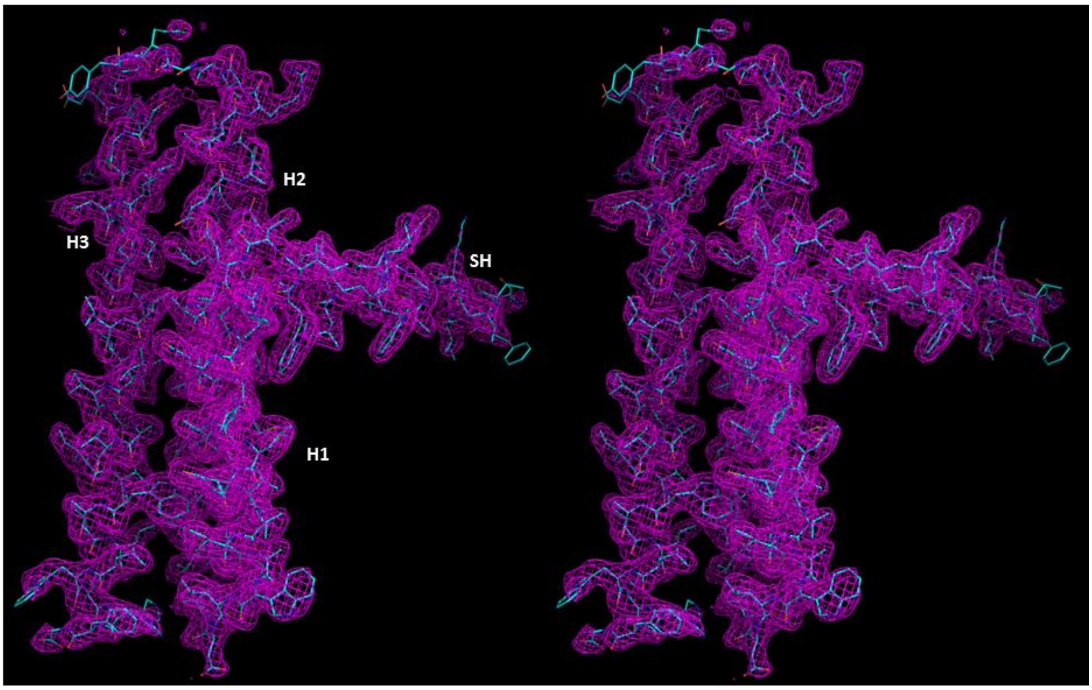

Supplementary Figure 2 | Stereo images of 2Fo-Fc electron density maps contoured at the 1 σ level of a single chain in structures reported in this paper. a, Δ4-99MAG-ACP. **b,** Δ4-99MAG. **c.** Δ7-79MAG-FEL. **d,** Δ7-79MAG. Details in Table 1.

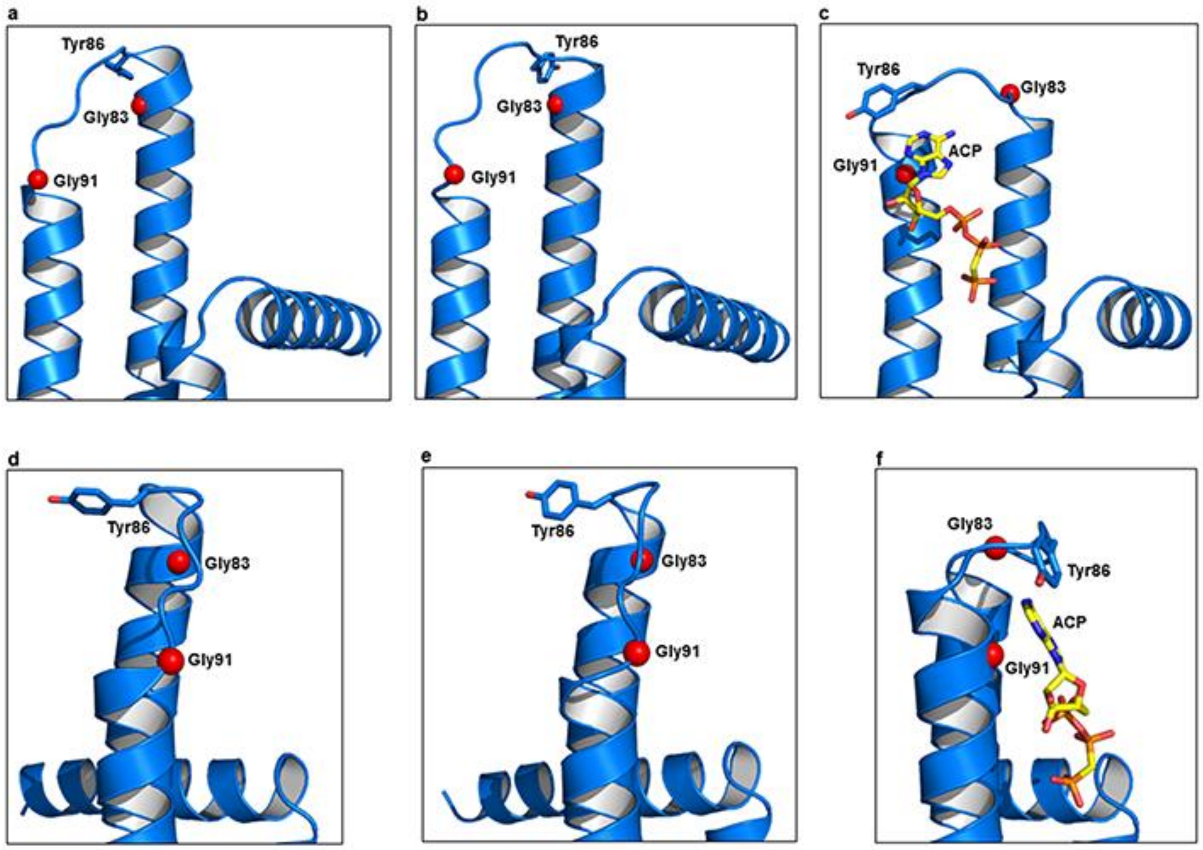

Supplementary Figure 3 | The three active sites in the DgkA ternary complex differ markedly in the cytoplasmic loop region. a, b, c, Views of the cytoplasmic loop in asCA, asAB and asBC, respectively, viewed from the cytosol and parallel to the membrane. ACP in asBC is shown in stick representation in **c**. Glycines are shown as red spheres. Residues referred to in the text are identified. In **a**, **b**, and **c**, the loop extends from Tyr86 to Gly91, Glu85 to Arg92, and Ile82 to Glu88, respectively. **d, e, f,** As in **a-c** rotated through ~90°. The hydroxyl group in the side chain of Tyr86 'moves' by ~17 Å in going from asCA to asBC.

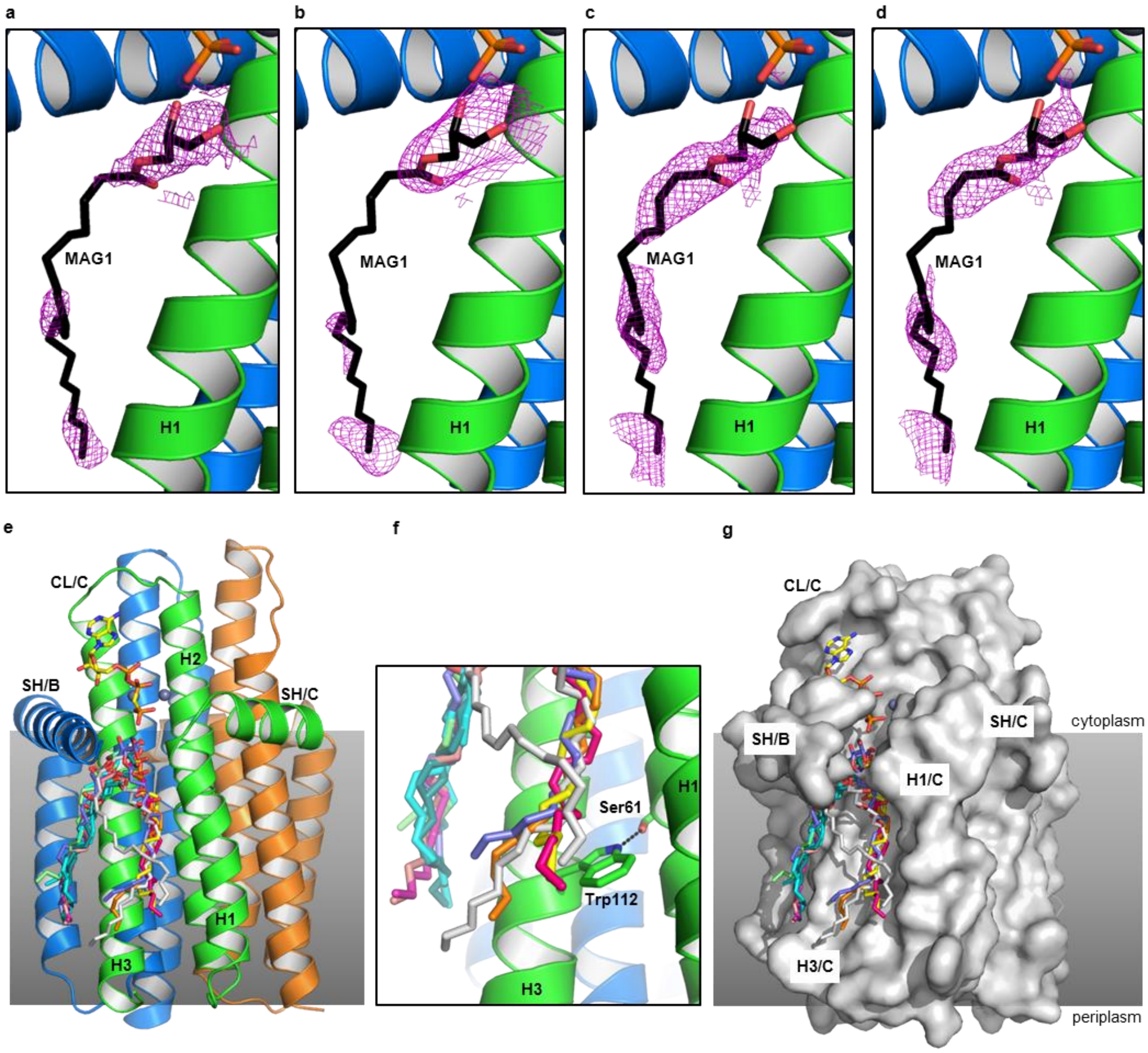

Supplementary Figure 4 | The lipid substrate binding site in DgkA. a, 2Fo-Fc electron density map for MAG1 in the ternary complex contoured at 0.7 σ. **b**, **c**, **d**, As in **a** with the electron density for MAG1 replaced by 2Fo-Fc density maps, contoured at 1 σ, observed in equivalent positions in Δ4-99MAG (**Table 1**), PDB ID: 3ZE3 and PDB ID: 4D2E, respectively. **e,** All of the MAGs found in the lipid substrate binding pocket of DgkA structures solved to date superimposed on active site asBC of the DgkA ternary complex. The collection includes 7.8 MAG, 7.9 MAG and 9.9 MAG from PDB ID: 3ZE3, 4D2E, and structures Δ4-99MAG-ACP, Δ7-79MAG-FEL, and Δ7-79MAG (**Table 1**). **f**, The base of the hydrophobic pocket accommodating the acyl chains of the lipid substrate is occupied by $Trp112_C$ which is held in place by hydrogen bonding to $Ser61_C$. **g,** Surface representation of the DgkA ternary complex model highlighting the hydrophobic pocket proposed to accommodate lipid substrate acyl chains. The same DgkA orientation is shown in **e** and **g**.

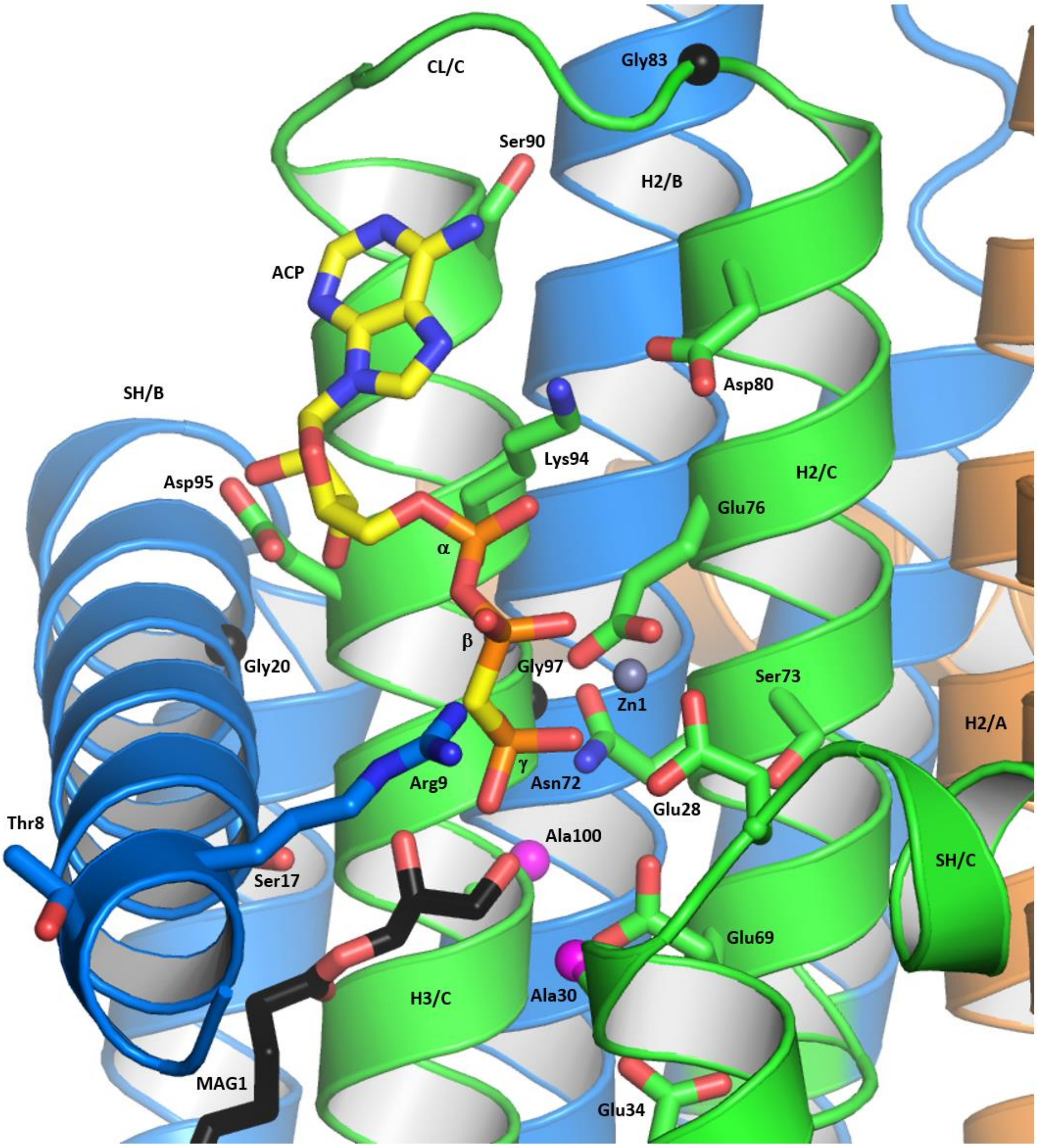

Supplementary Figure 5 | Functionally important residues mapped onto the ternary complex structure demark the active site of DgkA. The view is into the asBC active site. Included are residues identified as critical to kinase activity in mutation studies reported here (**Fig. 2b, Supplementary Table 1**). Electron density was not observed for the side chain of Arg9 and Glu28. The locations of glycines and alanines are marked with black and purple spheres, respectively.

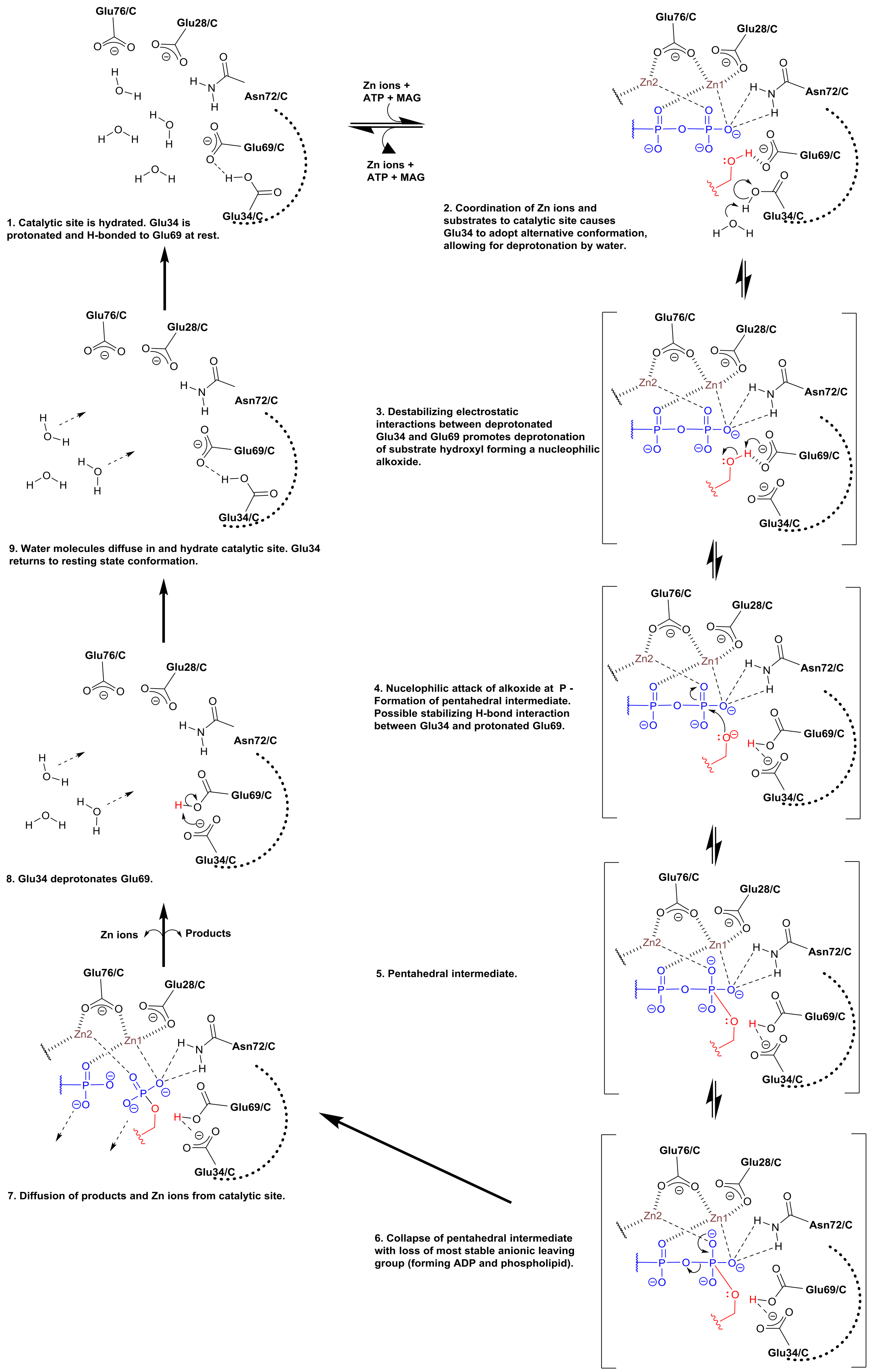
Zn ions +
ATP + MAG
Zn ions +
ATP + MAG
1. Catalytic site is hydrated. Glu34 is protonated and H-bonded to Glu69 at rest.
2. Coordination of Zn ions and substrates to catalytic site causes Glu34 to adopt alternative conformation, allowing for deprotonation by water.
3. Destabilizing electrostatic interactions between deprotonated Glu34 and Glu69 promotes deprotonation of substrate hydroxyl forming a nucleophilic alkoxide.
4. Nucelophilic attack of alkoxide at P - Formation of pentahedral intermediate. Possible stabilizing H-bond interaction between Glu34 and protonated Glu69.
5. Pentahedral intermediate.
6. Collapse of pentahedral intermediate with loss of most stable anionic leaving group (forming ADP and phospholipid).
7. Diffusion of products and Zn ions from catalytic site.
Zn ions
Products
8. Glu34 deprotonates Glu69.
9. Water molecules diffuse in and hydrate catalytic site. Glu34 returns to resting state conformation.
Glu76/C
Glu28/C
Asn72/C
Glu69/C
Glu34/C
Zn1
Zn2

Supplementary Figure 6 | Mechanism by which DgkA is proposed to catalyze the ATP-dependent phosphorylation of mono- and di-acylglycerols. This is an expanded, step-by-step version of the mechanism presented in **Fig. 2a**. Step 2 depicts $Glu34_c$ flipping to an alternative conformation which was observed in the XFEL structure data. This conformation could allow for deprotonation of $Glu34_c$ leading to destabilizing electrostatic interactions that enhance the basicity of $Glu69_c$, favouring deprotonation of the primary hydroxyl group of bound substrate. Deprotonated $Glu34_c$ may subsequently stabilize protonated $Glu69_c$. Upon product release the active site is then reset by $Glu34_c$ deprotonating $Glu69_c$. Square brackets denote short-lived intermediates.

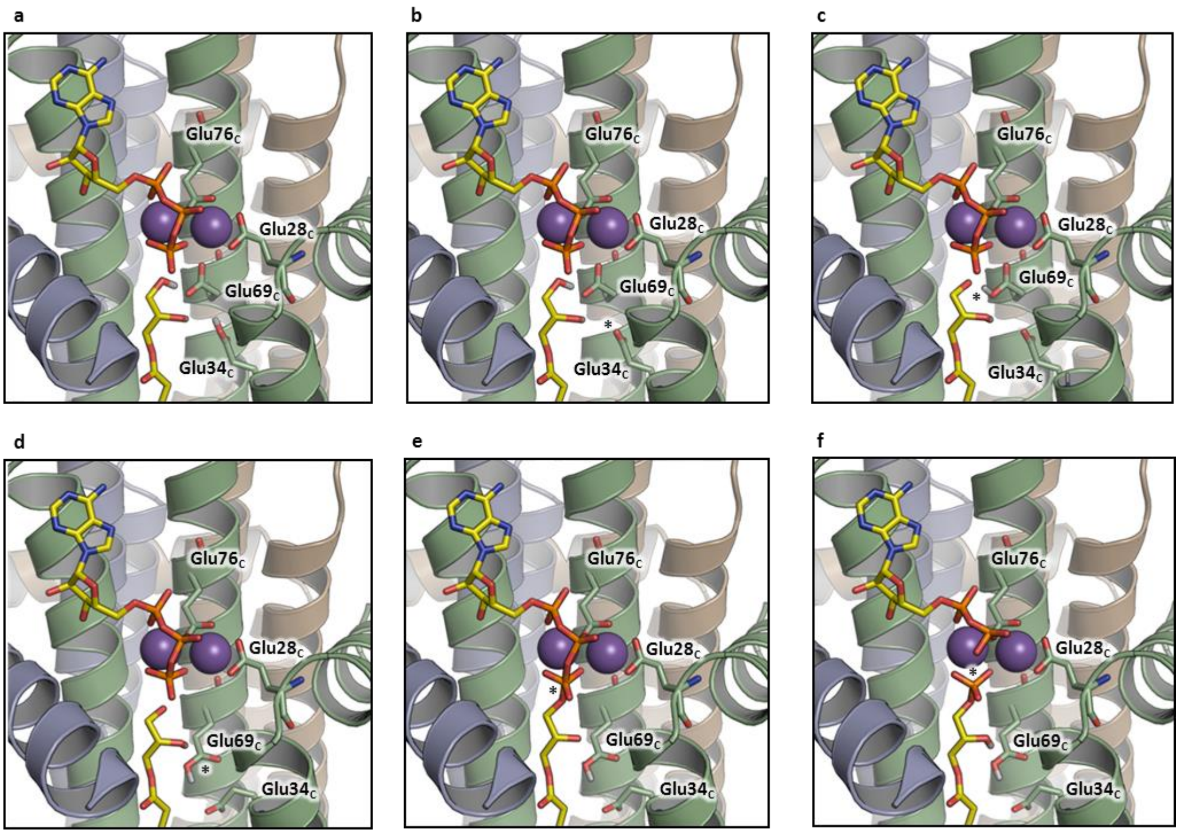

Supplementary Figure 7 | Modelling a mechanism from the DgkA structure. a, DgkA with ATP and MAG. The MAG primary hydroxyl is hydrogen bonded to Glu69$_C$. Glu34$_C$ is protonated and hydrogen bonds with Glu69$_C$. **b,** Substrate binding induces Glu34$_C$ to deprotonate. **c,** In response to deprotonation of Glu34$_C$, Glu69$_C$ strips the proton from the primary hydroxyl of MAG to create the alkoxide intermediate. **d,** Upon protonation, Glu69$_C$ changes its conformation to that observed in the XFEL structure and MD simulations. This is stabilized by donating a hydrogen bond to Glu34$_C$, which also adopts a second rotameric conformation. **e,** The deprotonated MAG performs a nucleophilic attack on the γ-phosphate of ATP to form a pentavalent intermediate. **f,** Products ADP and lysophosphatidic acid. Glu34$_C$ reprotonates from Glu69$_C$ to reset the DgkA to the state observed in **a**. Points of note in individual panels are annotated by an asterisk.

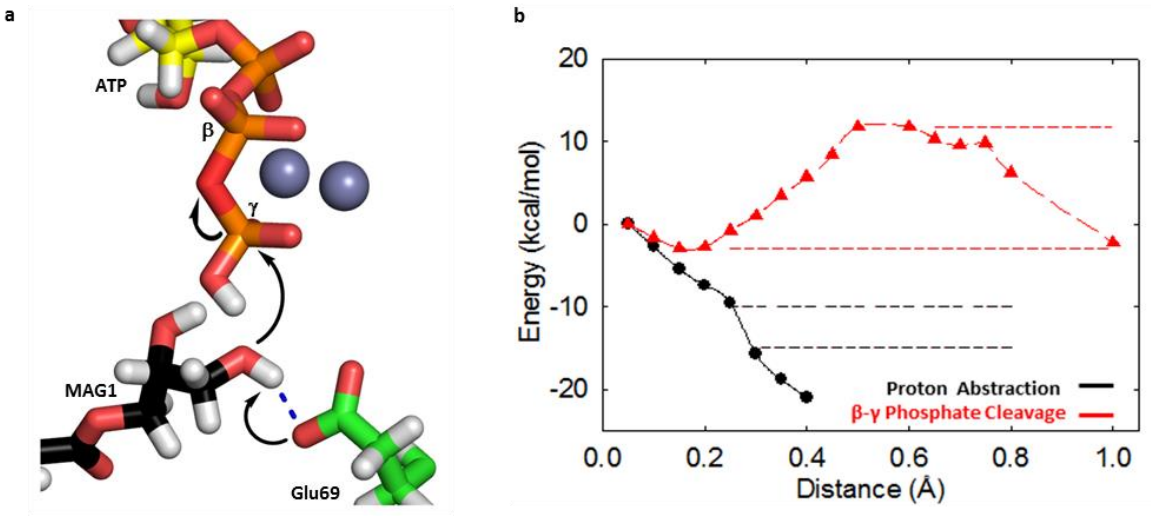

Supplementary Figure 8 | DFT simulations suggest that the rate limiting step in the kinase reaction is phosphate cleavage, not proton abstraction. DFT on MDS configurations displaying intimate MAG1 contacts (similar to **Fig. 3c**) reveals that 1-OH proton abstraction by $Glu69_C$ is more kinetically accessible than phosphate cleavage, in support of the proposed mechanism (**Supplementary Fig. 6**). This suggests the latter step is rate-limiting, in accord with $Asn72_C$ and/or $Arg9_B$ stabilizing the pentavalent intermediate (**Fig. 2a**), and MDS evidence of significant Arg9-mediated stabilization. **a**, Enlargement of the active site, with pathways indicated for both proton transfer between 1-OH and the Glu69 residue and cleavage of the bridging γ-P-O bond. Note that the ACP in the complex structure has been replaced in this image by ATP to reflect that actual substrate. **b**, Relative potential energy profiles for the proposed proton abstraction from 1-OH by Glu69 and phosphate cleavage steps indicated in **a** (with respect to the proton being 0.05 Å from its initial position, still 'bound' covalently to the O atom in 1-OH, and to 'stretching' of the bridging γ-P-O bond from its original length). The profiles were computed from calculations along both paths, moving the proton or extending the P-O bond in 0.05-0.1 Å steps. The left of each plot at zero distance denotes the 'starting point'. The distinct change of slopes (marked by horizontal dashed lines) indicate intermediate, transitional structures, in "no man's land", wherein the original covalent bond is ruptured. For proton abstraction, the proton has moved ~0.25-0.3 Å from 1-OH's oxygen atom and that O-H covalent bond ruptures, whilst by ~0.4 Å away, it is now bound to Glu69. For phosphate cleavage, the original bond ruptures at extension of ~0.5-0.6 Å. The 'kinks' in slope in and around these intermediate structures provide overestimates of the energy barrier.

Supplementary Table 1. Sensitivity of kinase activity to mutation and residue conservation in DgkA.

Construct [a]	Activity, % [b]	Activity of Cys mutant, % [c]	Substitutions tolerated [d]	Conservation, % [e]
WT	100			
T8A	4	0	I, P, R, V, S, A, N	31, K(25), R(14)
R9A	11	1.5	H, A, G, C, R, V, S	91, H(5)
R9K	19			
R9H	7			
R9E	12			
A13K	92	7	V	94, H(4)
A13R	24			
A13L	71			
S17A	15	3	C, A	98, T(1)
G20A	1	0.3	V	>99, A(<1)
E28A	8	6	R, D, A	100
E28D	8			
E28Q	4			
E28N	9			
E28R	0.1			
A30L	7	2	S, G, V, P	94, S(6)
R32A	48	1	G	>99, Q/K(<1)
R32K	30			
E34A	25	7	D, A, Q, G, V	88, L(8)
E34D	37			
E34Q	25			
E69A	0.2	0	None	100
E69Q	0.1			
E69D	0.2			
N72A	0	0.4	S	100
N72Q	3			
N72D	2			
S73A	19	4	C, A, T	92, T(8)
E76A	0	0.4	None	100
E76D	0			
E76Q	0			
D80A	12	2	E, N	94, E(5)
D80E	26			
D80N	43			
R81A	32	0.8	G, S, H	97, H(1)
R81K	44			
G83P	0.2	17	V, A, E	71, S(28)
Y86A	89	62	S, N, F	13, H(23), R(22)
Y86F	84			
S90P	0.2	10	C, G	80, A(18)
K94A	0.8	2.6	None	100
K94M	2			
K94R	0.5			
D95A	2	0.2	None	>99, N(<1)
D95N	95			
D95E	24			
G97P	0	5	S, C, A	91, A(8)
S98A	79	0.5	A, C	>99, A(<1)
A100L	0.4	6	G, S, C	97, S(1)
W112		84	Q, C	>99, H(<1)

[a] Construct is identified by residue identity and sequence number in wild-type DgkA followed by identity of the substituting residue. [b] This work. 100% activity corresponds to the rate recorded with wild-type DgkA *in meso* of 21 μmol min^{-1} mg^{-1}. [c] Data taken from Ref[1] where the effect of site specific mutations to Cys on the kinase activity of wild-type DgkA in detergent micelles was measured. % values reported are relative to wild-type. [d] Data taken from Ref[2] where each residue was mutated randomly to all possible amino acids. Mutations with greater than 5% kinase activity compared to wild-type are listed. Note that, in the latter study, each mutation was usually accompanied by mutations at other sites in the protein. This

might explain why substitution to Cys (bold red C in Column 4) at certain locations in the sequence are reported as tolerated where, according to the data from Sanders et al. in Column 3, such mutations significantly reduced kinase activity. [e] Sequence homologues were identified by performing a BLAST[3] search of the *E. coli* DgkA sequence against the Uniref100 sequence database[4] on the Uniprot server, thereby considering only unique DgkA sequences (1,000 in total). Sequences were aligned using Clustal Omega[5]. Conservation percentages were calculated using locally written code. In cases where conservation was not 100%, alternative residues are listed along with percent frequency of occurrence in brackets.

Supplementary References

1. Van Horn, W. D. *et al.* Solution nuclear magnetic resonance structure of membrane-integral diacylglycerol kinase. *Science* **324**, 1726-1729 (2009).
2. Wen, J., Chen, X. & Bowie, J. U. Exploring the allowed sequence space of a membrane protein. *Nat. Struct. Biol.* **3**, 141-148 (1996).
3. Altschul, S. F., Gish, W., Miller, W., Myers, E. W. & Lipman, D. J. Basic local alignment search tool. *J. Mol. Biol.* **215**, 403-410 (1990).
4. Suzek, B. E., Huang, H., McGarvey, P., Mazumder, R. & Wu, C. H. UniRef: comprehensive and non-redundant UniProt reference clusters. *Bioinformatics* **23**, 1282-1288 (2007).
5. Sievers, F. *et al.* Fast, scalable generation of high-quality protein multiple sequence alignments using Clustal Omega. *Mol. Syst. Biol.* **7**, 539 (2011).
